# The Relationship Between Work-Related Stress and Depression: A Scoping Review

**DOI:** 10.3389/phrs.2024.1606968

**Published:** 2024-05-01

**Authors:** Jean-Baptist du Prel, Adrijana Koscec Bjelajac, Zrinka Franić, Lorena Henftling, Hana Brborović, Eva Schernhammer, Damien M. McElvenny, Eda Merisalu, Nurka Pranjic, Irina Guseva Canu, Lode Godderis

**Affiliations:** ^1^ Department of Occupational Health Science, University of Wuppertal, Wuppertal, Germany; ^2^ Institute for Medical Research and Occupational Health, Zagreb, Croatia; ^3^ University of Zagreb, School of Medicine, Andrija Štampar School of Public Health, Zagreb, Croatia; ^4^ Department of Epidemiology, Center for Public Health, Medical University of Vienna, Vienna, Austria; ^5^ Research Group, Institute of Occupational Medicine, Edinburgh, United Kingdom; ^6^ Centre for Occupational and Environmental Health, University of Manchester, Manchester, United Kingdom; ^7^ Estonian University of Life Sciences, Tartu, Estonia; ^8^ Department of Occupational Medicine, Faculty of Medicine, University of Tuzla, Tuzla, Bosnia and Herzegovina; ^9^ Department of Occupational and Environmental Health, Unisanté, University of Lausanne, Lausanne, Switzerland; ^10^ Department of Primary Care and Public Health, University of Leuven, Leuven, Belgium; ^11^ IDEWE, External Service for Prevention and Protection at Work, Heverlee, Belgium

**Keywords:** psychosocial work stress, work-related depression, depressive symptoms, effort-reward imbalance, job strain

## Abstract

**Objectives:**

Work-related stress is highly prevalent. Recent systematic reviews concluded on a significant association between common work-related stress measures and depression. Our scoping review aims to explore whether work-related psychosocial stress is generally associated with depression or depressiveness, the extent and methodology of the primary research undertaken on this topic and to elucidate inconsistencies or gaps in knowledge.

**Methods:**

We searched for literature in Pubmed, PsycInfo and Web of Science including full reports in seven languages published between 1999 and 2022 and applied the PRISMA statement for scoping reviews criteria.

**Results:**

Of 463 primarily identified articles, 125 were retained after abstract and full-text screening. The majority report significant associations between work-related stress and depression. Cross-sectional studies are most prevalent. Sufficient evidence exists only for job strain and effort-reward imbalance. Most studies are from Asia, North America and Europe. The health sector is the most studied. Several research gaps such as the lack of interventional studies were identified.

**Conclusion:**

The consistency of most studies on the significant association between work-related stress and depression is remarkable. More studies are needed to improve evidence and to close research gaps.

## Introduction

Work-related stress is highly prevalent. In 2019 38 percent of workers globally reported experiencing high daily stress [1]. Occupational stress can be associated with absenteeism, presenteeism, low productivity or early work exits [2–6]. Moreover, occupational injuries can be associated with work-related stress [7]. Approximately 363,000 occupational fatalities and 26 million DALYs were caused by occupational injuries worldwide in 2016 [8]. For Europe, Australia and North America the annual costs of work-related stress to society were estimated between 221 million to 187 billion U.S. dollars, 70%–90% of which was caused by loss of productivity [9].

Apart from physical load, psychosocial factors can be a considerable source of stress at work. Half the workers in industrialized countries judge their work to be “mentally demanding” [10]. The working environment and the individual characteristics are fundamental in the development of work-related psychosocial stress [11]. According to the transactional theory of stress, the reaction to an environmental stimulus depends on the individual’s appraisal of it as challenging or threatening [12, 13]. The individual response to a stressful event can be different depending on the person’s coping strategy [14]. Health-related risks associated with chronic stress exposure can vary with genetic predisposition, epigenetic changes due to stressful early life experiences, and medical preconditions [15–17]. Work stress can influence employees’ health via a primary stress reaction with mediators (e.g., cortisol) and allostatic load affecting the cardiovascular, metabolic and immune systems and the brain [18, 19] or can lead to risky health behaviours (e.g., smoking or alcohol consumption) [20, 21]. Work-related stress is a risk factor for coronary heart disease [22] and type 2 diabetes mellitus [23]. Moreover, chronic stress exposure can impair mental health, with mild to severe subjective symptoms, burnout, psychosomatic and psychiatric diseases such as depressive disorders [7, 24–27].

Depressive and anxiety disorders are by far the most common mental disorders [28]. Approximately 12 billion working days are lost annually due to depression and anxiety, associated with cost of US$ 1 trillion by loss of productivity, globally [29]. In 2019 there were worldwide 279.6 million people (95% CI: 251.6–310.3) suffering from depression, which was 1.56-fold more frequent in women than in men [28]. Apart from significantly reduced life expectancy in both men and women after early onset of the depression [30], the occurrence and persistence of depressive symptoms significantly impact working life and the working environment. The estimated number of future employment years at the age of 30 for workers experiencing high depression symptoms throughout their working life, is more than 15 years shorter than for workers experiencing persistent low depression symptoms [31].

An imbalance between work demands and resources can contribute to work-related psychosocial stress [32–34]. About 30% of workers are in jobs with higher job demands than job resources in Europe, with between-country variation [35]. Health (45%), transport (42%) and agriculture (40%) are the sectors with the highest proportions of stressful jobs [35]. While this sector-specific prevalence of work-related stress is known, an overview of studies investigating the association between work-related stress and depression in different occupations is missing. Furthermore, it would be interesting to know which measurements for work-related stress and depression were used in studies investigating their association.

Often stress measurements are based on theoretical models. Two of the most investigated stress models, the job demand-control (JDC) model of Karasek [32] and the effort-reward-imbalance (ERI) model of Siegrist [33], and extensions of these, such as the job-demands-resources-model of Bakker and Demerouti [34], assume an imbalance between job demands and resources. Definitions of the latter differ depending on the stress model used. For instance, job resources can be non-monetary like decision latitude, a part of the JDC model, or monetary like wages, a part of the ERI model. Systematic reviews and meta-analyses have provided epidemiological evidence that work-related stress measured with ERI [26] and JDC [25, 27, 36] is associated with depressive symptoms or clinical depression. Yet, a comprehensive overview of the current state of research on the association between any measure of work-related stress and depression or depressiveness including research gaps and inconsistencies in knowledge is missing.

The aim of this scoping review of studies including data from 1999 to 2019 is to explore whether work-related psychosocial stress is associated with depression or depressive symptoms in general and to elucidate the research questions mentioned above:

1. Which occupational groups were most frequently investigated in the included studies on the association between work-related stress and depression or depressiveness?
2. Which measuring instruments of work-related psychosocial stress, as a whole or by individual components, were used in the identified studies?
3. Which measuring instruments of depression or depressive symptoms were used in the identified studies?
4. What knowledge exists on the relationship between work-related psychosocial stress and depression or depressiveness, in the identified studies?
5. What are the inconsistencies or gaps in knowledge regarding the factors contributing to the relationship between work stress and depression or depressiveness?

## Methods

### Protocol and Registration

This scoping review performed in accordance with the PRISMA Extension for Scoping Reviews guidelines [37] is based on a protocol registered at the Open Science Framework under registration number hg7r4-v1 [38].

### Databases Used

A literature search was performed by two independent search teams in PubMed, Web of Science Core Collection and PsycInfo using predefined search strings and filters (Supplementary Table S1).

### Eligibility Criteria

Primary research articles published between January 1999 and April 2022 in the form of full reports, focusing on the association of psychosocial work-related stress as the exposure and depression or depressive symptoms as the outcome measured with a questionnaire or a diagnostic interview were included. Only studies which finished data collection before December 2019 were considered, to avoid bias by any changes in the work environment introduced by the ensuing SARS-CoV 2 pandemic. We considered all forms of interventional and observational studies but no reviews, qualitative studies, abstracts, letters to the editor or commentaries. Only studies with employees, but not employers or managers were included, to focus on harmonised study groups. Publications in English, German, Bosnian, Croatian, French, Italian, Serbian and Serbo-Croatian were included according to the researchers’ language fluencies. Excluded were pharmacological studies, studies with unemployed persons or those focusing on physical stressors. Studies with mental comorbidities (except anxiety as a symptom of depressiveness) were excluded to avoid erroneous appraisal of the work-related stress effect on depression or depressiveness. The electronic search strategy can be found in Supplementary Table S1 in the online Supplementary Material.

### Study Selection

**FIGURE 1:**
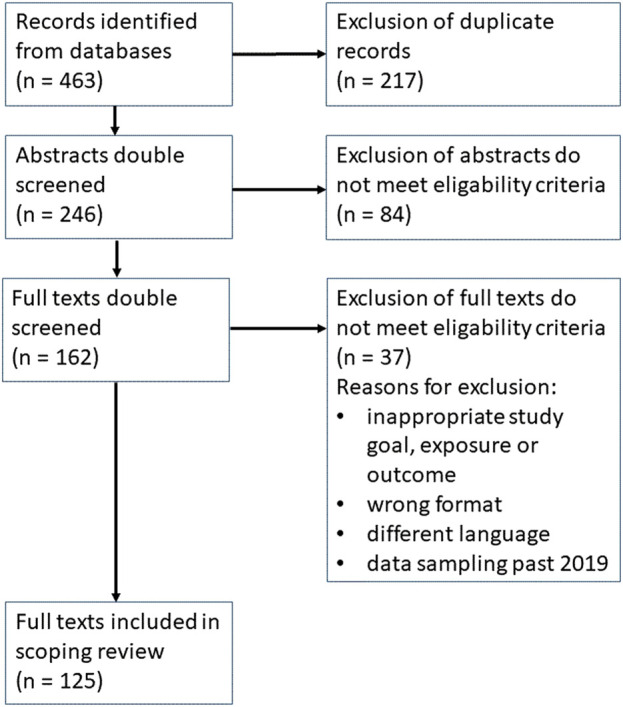
Identified studies in Pubmed, PsycInfo and Web of Science (scoping review on the relationship between work-related stress and depression, six continents, 1999–2022).

Duplicates found in different literature data basis were excluded. The screening of studies found in the literature research followed two steps (Figure 1). First, titles and abstracts were independently screened by two reviewers against the inclusion and exclusion criteria. Then, full texts of the remaining articles were screened for inclusion and exclusion criteria by two reviewers. In both steps a third reviewer helped get agreement about in- or exclusion of articles when necessary.

### Data Extraction

A specially developed data extraction form was used by the reviewers to independently extract the data from eligible studies. Relevant information on key study characteristics and detailed information on all metrics used to estimate/describe work-related stress, depression/depressiveness and relevant information about covariates. The data-extraction process was conducted twice. Any inconsistencies and disagreements were resolved through discussion between the reviewers or with a third reviewer before transfer of results in Supplementary Table S2.

Finally, the study characteristics were reported in Supplementary Table S2, the number of studies per country and continent in Table 1 and the frequencies of the used exposure and outcome measurements in Supplementary Tables S3, S4.

**TABLE 1:** Included studies per continent and country (scoping review on the relationship between work-related stress and depression, six continents, 1999–2022). (N = 125).

Continent (no) [%]	Country (no) [publication]
Asia (57) [45.6%]	CHN (15): [39–53]; ISR (1): [54]; JPN (24): [55–78]; KOR (9): [79–87]; MYS (1): [88]; PHL (1): [89]; TUR (1): [90]; TWN(5): [91–95]
Europe (37) [29.6%]	BEL(1): [96]; DEU (14): [97–110]; DNK (2): [111, 112]; FIN (5): [113–117]; FRA: (2): [118, 119]; GBR (2): [120, 121]; SWE (7): [122–128]; UKR (1): [129]; Different (3): [130–132]
North America (20) [16.0%]	CAN (5): [133–137]; USA (15): [138–152]
Oceania (4) [3.2%]	AUS (3): [153–155]; NZL(1): [156]
Africa (3) [2.4%]	EGY (2): [157, 158]; GHA (1): [159]
South America (1) [0.8%]	BRA (1): [160]
Different (3) [2.4%]	[161–163]

AUS, Australia; BEL, Belgium; BRA, Brasilia; CAN, Canada; CHN, China; DEU, Germany; DNK, Denmark; EGY, Egypt; FIN, Finland; GBR, United Kingdom; GHA, Ghana; ISR, Israel; JPN, Japan; KOR, South-Korea; MYS, Malaysia; NZL, New Zealand; PHL, Philippine; SWE, Sweden; TUR, Turkey; TWN, Taiwan.

### Synthesis of Results

Results were summarized narratively. We grouped the studies by 1) occupation, 2) types of stress measurement, 3) measurement of depression/depressiveness and 4) study type, and summarized the type of settings, populations and study designs for each group, along with the measurements used and broad findings.

## Results

Of 463 articles identified in the literature searches after exclusion of duplicates and those not meeting the inclusion criteria in the title, abstract and full text screening, 125 studies [39–163] were included in the data extraction process (Figure 1).

The studies selected for analysis are charted in Supplementary Table S2. This provides information about study type and design (prospective or retrospective), continent and country, occupation, number of subjects, sociodemographic characteristics, occupational stress measure as the exposure, time of exposure measure, depression (categorized) or depressiveness (continuous) as the outcome, effect size of the association between exposure and outcome (incl. *p*-values and confidence intervals), factors adjusted for, a short summary of key findings and remarks when needed.

### Study Characteristics

Among the articles included, 67.2% were cross-sectional studies, followed by cohort studies (20.0% prospective, 1.6% historical), other longitudinal studies (7.2%), interventional studies, including one RCT (2.4%), one case-control-study (0.8%) and one clinical non-interventional study (0.8%) (Supplementary Table S2).

### Occupational Groups

Analysed studies included healthcare workers (24.0%), manufacturing workers (7.2%), education/teachers (4.0%), public servants (4.0%), emergency workers (2.4%), farmers and fishermen (2.4%), clergy (1.6%) and managers/executives (1.6%). Most studies (36.0%) included employees from different occupations (Supplementary Table S2).

Nurses, nearly all female, were the most common studied occupational group in 16 studies, with composite measurements or singular components of work stress showing a significant association with depression. Physicians (14 studies) were the second most frequent group, with the ERI questionnaire being the most frequently used stress measurement (42.9%). 85.7% of doctors’ studies found significant associations between work-related stress and depression or depressiveness. Support from co-workers, working shorter hours and not working at weekends may lead to a risk reduction [67, 109].

Manufacturing workers were represented with nine studies, all but one [138] conducted in Asia. Five studies focused on teachers and public servants, three studies on emergency workers and farmers/fishermen and two on clergy and managers/executives. The remaining 66 studies addressed occupations by only a single study (12), different occupations in one study (45) or the occupations were not stated (9).

Overall, beside nurses, physicians and manufacturing workers the number of studies investigating this association in other occupations is limited.

### Measuring Instruments of Work-Related Psychosocial Stress

Measurement instruments of work-related psychosocial stress were most frequently based on Karasek’s JDC model [32]: seventeen studies used the JDC questionnaire, 10 of these without, and seven including the support component (JDC-S). Different versions of the Job Content Questionnaire (JCQ) were used in 22 studies, half of them with and half without the support component. Other instruments related to JDC were the Brief Job Stress Questionnaire (BJSQ) (7), the Child Care Worker Job Stress Inventory (CCW-JSI) (2), the Job Stress Scale (JSS) (1), the Brief Stress Scale (BSS) (1), the Work Stress Scale (1), the Copenhagen Psychosocial Questionnaire (2), the Shortened Stress Evaluation Tool (1), and the Psychosocial Leave-Behind Questionnaire (1) (Supplementary Table S3).

Second most common (26.4%) were stress measurements based on Siegrist’s ERI model [33]. Eight studies applied the original, 14 studies long and 11 studies short versions of the ERI-questionnaire. Twenty studies included measurements of the extrinsic (efforts and rewards) and the intrinsic component (overcommitment) of the ERI model and 13 studies the extrinsic component alone. Thirteen studies combined ERI components with other stress measurements. Furthermore, country-specific and modified versions of the ERI-instrument were often used.

7.2% of studies used the Generic Job Stress Questionnaire developed by the US National Institute for Occupational Safety and Health (NIOSH-GJSQ) [164]. The Korean Occupational Stress Scale (KOSS) [165] (6.4%) includes items from the JCQ, ERI and NIOSH-GJSQ. One study [47] applied the Job Burden-Capital Matching Model [166] which combines questions from the JCQ and ERI. Thirty-eight studies used a variety of stress assessment tools including perceived stress measures [167, 168], occupation specific instruments [169–173] and instruments focused on certain stressful aspects like workplace bullying [174]. One study [128] used a Job-exposure-Matrix (JEM), including job demand and control items to classify jobs, with respect to the stress prevalence and identify those corresponding to a stressful work environment. JEM is a widely used exposure assessment tool in occupational epidemiology, especially when individual exposure measurement data are unavailable [175].

Altogether, the majority of studies used stress measurements based on the JDC- and ERI-model or components of them followed by the NIOSH-GJSQ.

### Measuring Instruments of Depression or Depressive Symptoms

The most common instrument to assess depression or depressive symptoms (n = 57) was the Center for Epidemiological Survey-Depression Scale (CES-D) [176], used in its original, or long, short, or modified version and in different languages. Sixteen studies used the Beck Depression Inventory (BDI) [177], and 12 studies the Patient Health Questionnaire (PHQ) [178]. Other commonly used tools include Zung’s Self-Rating Depression Scale [179], EURO-D depression scale [180] and the Hopkins Symptom Checklist [181], to name only a few. Five studies used ICD-9 or ICD-10 diagnoses; three studies relied on DSM-IV Diagnosis. Only three studies created *ad hoc* scales. One of them a 5-item scale assessed feelings over the past 4 weeks. Another one assessed two domains of depression in the past 2 months (Supplementary Table S4).

In summary, most of the studies applied validated measurements of depression or depressiveness. CES-D, BDI or PHQ were most commonly used.

### Existing Knowledge on the Relationship Between Work-Related Psychosocial Stress and Depression or Depressive Symptoms

High job strain (high job demands, low control) as measured by JDC, JDC-S, JCQ, JSS, KOSS, BJSQ, CCW-JSI or Job Burden-Capital Matching Model was associated with depressiveness or depression in most of cross-sectional and longitudinal studies. Yet, in some studies only components of the JDC-model (e.g., control) were significantly associated with depression or depressiveness cross-sectionally [39, 76, 81, 97, 143, 151] or longitudinally [115, 117, 131, 138, 143, 152, 161, 162]. Social support was negatively associated with depressiveness in one cross-sectional study [141], but not in another [48] nor in two cohort studies [73, 126] (Supplementary Table S2).

Effort-reward ratio (ERI-R) [182], or components of the ERI-model were significantly associated with depressiveness or depression in most cross-sectional and all longitudinal studies. In one cross-sectional study the significant association disappeared after adjustment for burn-out [53]. Two studies found the strongest association either with personal [102] or organisational rewards [77]. Both found monetary rewards to be the weakest. The length of working hours was also found to affect the association [74, 109]. A longitudinal study found comparable strengths of association between ERI and depression for women and men [107], another observed a significant association between ERI and depressiveness over time in Europe, but not in the United States or Japan [162]. An interventional study found that reducing ERI-stress through interpersonal psychotherapy was more effective than standard therapy [108]. Overcommitment, independent of the extrinsic component of the ERI-model, has been linked to depression or depressiveness cross-sectionally [44, 46, 71, 74, 76, 98–100, 103, 155] and longitudinally [101, 107, 118]. Some studies found a significant bivariate association without controlling for confounders [40, 102, 119]. Studies combining components of ERI and JDC independently have shown an association with depressive symptoms or depression cross-sectionally [76, 98, 136] and longitudinally [130, 144, 161].

Most cross-sectional studies [55, 56, 65, 66, 69, 74, 78] and a longitudinal study [57] found dimensions of the NIOSH-GJSQ, such as role ambiguity or low job control associated with depression or depressive symptoms. Most studies using KOSS [79, 80, 82–85, 87] found at least one component significantly associated with depression or depressiveness. Less often used stress assessment tools were also significantly associated with depressiveness or depression in cross-sectional [41–43, 48–50, 52, 59, 60, 62, 80, 91, 92, 94, 110, 139–141, 145, 146, 148, 149, 157, 163] and longitudinal studies [137, 142, 150] (for effect strength of the association under review see Supplementary Table S2).

#### Region

The majority of studies were conducted in Asia, Europe and North America, less frequent were studies from Oceania, Africa, South America or transcontinental regions. Over half were conducted in Japan, United States, China and Germany (Table 1). A cross-country comparison between United States, Europe and Japan [162] found for ERI and low job control a significant cross-sectional link to depression except low control in Japan. Significant longitudinal associations were found for these stress measurements only in Europe (Supplementary Table S2).

#### Gender

A study found a stronger association between work-related stress (JCQ) and depression for female employees in public administration [96], while male automotive manufacture workers with higher socioeconomic status were more vulnerable to interpersonal conflict [56]. Job demands were significantly associated with depression only in men, both cross-sectionally [84, 127] and longitudinally [54]. Conversely, organizational injustice and low influence at work were risk factors and support from superiors and fellow workers protective factors only in females [84, 112, 127]. In other studies, job strain [72, 124] and demands [68, 87] were significantly associated with depressiveness or depression in both genders, with a stronger effect in men. Job control [87, 112] and job security [87] were significant risk factors for depression only in men and organizational injustice only in women [87]. The association between the ERI-ratio and depressive symptoms was stronger in men in Germany [100], but no significant gender-specific differences in this association has been observed in Europe longitudinally [107]. A bidirectional longitudinal association between work-related stress measured by workload and job control and depressiveness has been shown in men [54].

#### Age

In older employees (50+) significant longitudinal associations between ERI [130, 132, 144, 161, 162] and job strain [130, 144] or control [161, 162] and depression and depressiveness, and cross-sectional evidence of this association for some other stress measurements [50, 149] were found. However, no studies focused on employees younger than 25, and while two studies showed a higher risk for depression in employees under 30, no age stratified analysis was found.

#### Mediators

Work-related psychosocial stress and depressiveness are linked through various mediators: Job strain [32] and depressive symptoms were mediated by burn-out cross-sectionally [53, 95, 114, 139], while longitudinal evidence is limited to dentists and two study waves [113, 115]. Sleep quality was another mediator in this association among psychiatric nurses [95]. For ERI and depressiveness mediating effects of psychosocial capital [44] and work-family conflict have been found cross-sectionally [100]. Moreover, ERI can partly explain the social inequality in depressiveness [99, 130].

#### Moderators

Social support, job autonomy, job satisfaction and job security can moderate (“buffer”) the negative effect of different job stressors on depression [39, 43, 68, 137]. Furthermore, an interaction between hair cortisol level and work stress (MSIQ) on depression in fishermen was observed [51]. Severe work-related stress may curtail as a moderator the protective effect of spirituality on depression [89]. Moreover, a reduction of work stress has been reported when reducing working hours [109].

Overall, most studies (n = 115) found a statistically significant association between work-related stress and depression or depressiveness, at least for some measurements, regardless of study type, period and region, occupational group, sociodemographic differences and the measuring instruments and form (self-reported or doctor’s diagnosis). The majority of studies are from Asia, Europe and North America. Some studies indicate that organizational injustice increases the risk of depression in women and job insecurity in men.

### Inconsistencies and Gaps in Knowledge

#### Sociodemographics

Studies on employees under the age of 25 on the association between work stress and depression are missing. Studies on gender-specific differences in this association are limited, with inconsistencies between studies using specific measures, e.g., regarding the protective effect of social support. More gender-specific or -stratified studies are needed to explain these differences.

#### Study Type

Only three interventional studies were identified: One was a stress-management programme [63] and another a workplace promotion programme [88], both for manufacturing workers. A third was a work-focused interpersonal psychotherapy programme for clinically diagnosed depression [108]. While all of these interventions were effective, interventional studies with more participants, different occupations and forms of stress interventions are needed.

#### Bias

One study concludes that reporting bias may inflate associations between high psychological demands and low decision latitude at work and the occurrence of depression [111]. Another suggests that there may be a substantial under-recognition and under-compensation of job strain-attributable depression [154].

#### Mediators and Moderators

More, especially longitudinal studies, are needed on factors mediating or moderating the association between work-related psychosocial stress and depressiveness or depression.

#### Measurement of Work-Related Psychosocial Stress

Studies show that various measures of work-related psychosocial stress contribute independently to depression [76, 98, 130, 136, 144, 161]. Reforms of mainstream models, like the job burden-capital model [155] integrate JDC, ERI and intrinsic personality factors. However, more investigation is needed to understand the independent influences of the different facets of psychosocial work-related stress on depression.

Altogether, gender specific inconsistencies exist in research on the association between certain work-related stress measures and depression. No studies were found for employees younger than 25. More interventional studies, research on bias, moderators and mediators of this association and studies on composite stress measurements are needed.

## Discussion

This scoping review provides new insights on the relationship between work-related stress and depression or depressiveness by including 125 studies from five continents over more than two decades. However, study data were only considered until the end of 2019 to avoid bias through additional stress or mood disturbances introduced by the SARS-CoV-2-pandemic [183]. Since the SARS-CoV-2-pandemic may have caused enduring changes in working conditions, this ought to be addressed in future studies.

We found large geographical differences in the state of research on this topic: Most studies came from Asia, Europe and North America, but only few from Oceania, Africa and South America. Transcontinental and transnational studies were scarce. Results on the association between work stress and depression, however, were surprisingly consistent regardless of geographic location. The healthcare sector has been most frequently investigated on this topic.

Consistent with an earlier review [36] we found Karasek’s job strain model [32] including instruments derived from it, to be the most commonly applied measure of work-related psychosocial stress followed by measuring instruments based on Siegrist’s ERI model [33]. CES-D was the instrument most commonly used to measure depression or depressiveness, followed by the BDI and the PHQ. Studies with a clinical diagnosis of depression were rare. The evidence for the association between job strain and ERI and depression seems to be sufficient given the high percentage of longitudinal and cross-sectional studies which found significant associations in line with meta-analyses [25–27]. Interestingly, studies which included components of job strain and ERI to measure work stress, found independent associations of both, with depression/depressiveness [76, 98, 130, 144, 161] indicating that both models identify different aspects of stressful work conditions. Different stress measurements may also be more or less appropriate for certain job types [76]. Under the high number of studies using other instruments to measure work stress than job strain or ERI only four longitudinal studies were identified [57, 140, 142, 150]. Nevertheless, the prevailing congruence in significant associations between psychosocial work stress and depression or depressiveness in cross-sectional and longitudinal studies using a variety of instruments to measure work stress and depression or depressiveness is remarkable. However, heterogeneity in results was found regarding the association of social support and depression regarding study types [73, 126, 141] and gender aspects [54, 112, 127].

Many studies have focused on specific occupational groups. It is warranted to consider potential limitations of this approach: The JCQ, which derives its cut-offs based on the respective population means [184], will be of limited usefulness if the range of job stress in a given occupational population is narrow (e.g., if all participants are working in nursing, with similar job demands and control). Associations may appear inflated in such studies, and would not be comparable to other occupational groups with different work stress exposures. Likewise, comparisons of study results on specific occupational groups with others can be difficult when using ERI-tertiles or -quartiles derived from the population under study [182]. We recognize the necessity to focus on specific occupational sub-groups to investigate specific job-related aspects in the association between work-related stress and depression and also for practical reasons. However, scientists should be aware of these methodological limitations when using stress measurements related to the population under study.

Furthermore, we acknowledge the importance of considering individual differences and contextual factors in explaining heterogeneity. While our review primarily focuses on psychological and sociological responses, integrating biological markers like allostatic load and epigenetics can offer a comprehensive understanding of the mechanisms underlying diverse responses to the phenomenon under study. By incorporating these multidimensional perspectives, future research can better elucidate the complex interplay between psychological, sociological, and biological factors in shaping individual variations in response.

One limitation of our scoping review is that psychometric tools used in some studies to assess depression have not been previously validated. However, when comparing results from these studies with others using validated depression instruments, we found largely similar results. Thus, a strength of this scoping review is to demonstrate the utility of these studies which may otherwise have been discarded based on their potentially weak outcome assessments.

Similarly, measurements of work stress varied greatly in our scoping review though the majority centred around two well established instruments (JDC and ERI). An interesting outcome of our review is that the association between work-related stress and depression was apparent, regardless of which measure used and which study type applied. Even though random misclassification in these unvalidated exposure assessments and publication bias cannot be ruled out, the consistency of this association makes it generally more probable in terms of plausibility.

Several research gaps of note were identified: Firstly, there is a lack of interventional studies, to investigate the effect of work stress alleviating interventions on depression prevention. Secondly, improved work-related stress assessment tools are needed incorporating different known and potentially novel stressors (e.g., dissolving boundaries at home office) in future studies. Our review focused on the pre-pandemic period as it is conceivable that pandemic-related changes could impact the association between work stress and depression. Nonetheless, our review aims to provide a reference basis for future studies. Moreover, some groups, such as younger workers are severely under-investigated. Presumably, because available measuring instruments of work stress do not include important stressful aspects (conflicts between work and school or study) and are created for full-time employment which is often not the case for younger employees [185]. Therefore, assessment tools focusing on stress items relevant to younger adults are required.

The strengths of our scoping review relate to the assessment of the association of work-related stress as the exposure, and depression as the outcome, irrespective of measurements used, or study designs implemented—providing for the first time a comprehensive picture of the existing literature. Another strength is its focus on the pre-pandemic time, alleviating any concerns regarding how work-related changes during the pandemic might have changed the work-stress profile of workers.

In conclusion, our results will serve as guidance for employers and employees alike to pay more attention to work stress given its impact on workers’ depression risk and its potential long-lasting consequences for the work ability of our future workforce. Moreover, the research gaps identified in this scoping review should be addressed in future studies.
