# Supplementary material for: The Relationship Between Work-Related Stress and Depression: A Scoping Review
Source: Public Health Rev. 2024 May 1;45:1606968. doi: 10.3389/phrs.2024.1606968 (PMC11094281; doi:10.3389/phrs.2024.1606968)
Supplement: Supplementary file 1 [file Table1.docx]

**Supplementary Table S1** **Literature Research in three literature data bases (Pubmed, PsychInfo, Web of Science*) on May 20, 2022, Scoping review on the relationship between work-related stress and depression** **(six continents, 1999-2022)**

| Search String/ Filters (Example: PsycInfo) | **Search Period** | | | | | |
| --- | --- | --- | --- | --- | --- | --- |
|  | **01/01/1999-31/12/2019** | | | **01/01/2020 – 20/05/2022** | | |
|  | **Pubmed** | **PsycInfo** | **WOS*** | **Pubmed** | **PsycInfo** | **WOS*** |
| TI ( (work stress OR work-related stress OR work related stress OR job strain OR occupational stress OR working stress OR professional stress OR job-related stress OR job stress OR work burden OR stress at work OR psychosocial work conditions OR psychosocial work environment OR job resources OR workplace stress OR work place stress OR effort-reward imbalance OR job control OR job demands OR job-related conditions) ) AND TI ( (depressive symptoms OR depressiveness OR depression OR depressive disorders OR depressive mood OR depressed mood OR dysthymia OR depressive disease OR depressive syndrome OR major depression OR clinical depression) ) AND TX stress NOT ( (coldness OR noise OR heat OR temperature OR wetness OR wind OR physical OR weather conditions) )  Publication Year: 1999-2019; Language: Bosnian, Croatian, English, French, German, Italian, Serbian, Serbo-Croatian; Population Group: Human; Document Type: Journal Article | 107 | 119 | 173 |  |  |  |
| TI ( (work stress OR work-related stress OR work related stress OR job strain OR occupational stress OR working stress OR professional stress OR job-related stress OR job stress OR work burden OR stress at work OR psychosocial work conditions OR psychosocial work environment OR job resources OR workplace stress OR work place stress OR effort-reward imbalance OR job control OR job demands OR job-related conditions) ) AND TI ( (depressive symptoms OR depressiveness OR depression OR depressive disorders OR depressive mood OR depressed mood OR dysthymia OR depressive disease OR depressive syndrome OR major depression OR clinical depression) ) AND TX stress NOT ( (coldness OR noise OR heat OR temperature OR wetness OR wind OR physical OR weather conditions) ) NOT TX (( covid or coronavirus or covid-19 or sars-cov-2 or coronavirus 2019 or pandemic ))  Publication Year: 2020-2022; Language: Bosnian, Croatian, English, French, German, Italian, Serbian, Serbo-Croatian; Population Group: Human; Document Type: Journal Article |  |  |  | 16 | 13 | 35 |

*Web of Science Core Collection (Arts & Humanities Citation Index, Science Citation Index Expanded and Social Sciences Citation Index)
