# Supplementary material for: The Relationship Between Work-Related Stress and Depression: A Scoping Review
Source: Public Health Rev. 2024 May 1;45:1606968. doi: 10.3389/phrs.2024.1606968 (PMC11094281; doi:10.3389/phrs.2024.1606968)
Supplement: Supplementary file 2 [file Table2.docx]

**Supplementary Table S2A Study characteristics, Scoping review on the relationsship between work-related stress and depression (six continents, 1999-2022)**

| **Author (year) + [Ref.]** | **Study type (direction)** | **Continent (Country)** | **Occupation** | **Subject number (Primary Response rate)** | **Age range AND/OR age mean (SD)^a^ [years]** | **Gender female** | **Other sociodemo-graphics** | **Exposure: Occupational stress measure**  **[stress scale] OR/AND [other measure]** | **Exposure measured in the present/past** |
| --- | --- | --- | --- | --- | --- | --- | --- | --- | --- |
| Ahola et al. (2007)  [113] | Other longitudinal study, prospective, | Europe (Finland) | Dentists | 2,555 (71%) | 26-73 | 74% | Marital status | Demand and control items of the Job Content  Questionnaire (JCQ) (Karasek et al., 1998) | Present |
| Ahola et al. (2006)  [114] | Cross-sectional study | Europe (Finland) | Full range of occupations | 3,270  (84%) | 30-64 | 48% | Marital status, occupational grade | Demand and control items of the JCQ | Present |
| Armon et al. (2010)  [54] | Longitudinal study (full panel), prospective | Asia (Israel) | Different | 692 (92%) | Working age | 32% | Seniority, marital status, number of children | Job Demands-Control-Support (JDC-S) questionnaire | Present |
| Bailey et al. (2015)  [156] | Prospective longitudinal study (“half longitudinal design”) | Oceania  (New Zealand) | Employees, randomly chosen | 1,081 (T1: 30.7%/T2: 31.2%) | 42.7 | 44.4% | Education, income, work status | Demand and control items of the JCQ | Present |
| Balog et al. (2003) [122] | Case-control study, retrospective | Europe (Sweden) | Different | CHD pt 292 (131 working)/control gr 300 (156 working)  70% CHD pt/63% controls | <65; mean age: 53,9 (±7) for cases, 54.5 (±7) for controls | 100% |  | Karasek‘s Job Demand-Control (JDC) questionnaire | Past |
| Batalla et al. (2018) [89] | Cross-sectional study | Asia (Philippine) | Nurses | 242 (80.7%) | 30.33 (±9.48) | 71.9% | Marital status, service length | Expanded Nursing Stress Scale (ENSS) | Present |
| Bernburg et al. (2016) [97] | Cross-sectional study | Europe (Germany) | Physicians (Internal Medicine, Neurology, Surgery, Paediatrics,  Anaesthesiology and Gynaecology & Obstetrics) | 435 (61.8%) | 27-57 | 51% | Years of working experience, marital status, presence of children | Copenhagen Psychosocial Questionnaire (COPSOQ); Perceived Stress Questionnaire (PSQ) | Past |
| Chen et al. (2009) [39] | Cross-sectional study | Asia  (China) | Manufacture workers | 483 (not reported) | <21 - >55 | 51.9% | Education level,  organizational types | A Shortened Stress Evaluation Tool (ASSET)- eight subscales of job stressors (work relationships, job nature, overload, job control, job security, resources/communication, work-life balance, pay and benefits), social support and coping | Present |
| Cho et al. (2008) [79] | Cross-sectional study | Asia (South Korea) | Workers in different industries | 8,522  (67.5%) | 21-65; 38.6 (±9.1) | 20.8 % |  | Korean occupational stress scale (KOSS) | Present |
| Clays et al. (2007) [96] | Cohort study, prospective (two waves) | Europe (Belgium) | Workers from public administration | 2,821 (67.2% 2nd phase (first not mentioned)) | 35-59 | 30.9% | Educational level | JCQ / JDC-S questionnaire | Present |
| Da Silva Gherardi-Donato et al. (2015) [160] | Cross-sectional study | South America  (Brazil) | Nursing technicians and nursing assistants | 310 (91.7%) | < 40 yrs: 28.7%;  ≥ 40 yrs.: 71.3% | 76.1% | Marital status, religious practice | Childhood Trauma Questionnaire (CTQ) for early stress exposure,  Job Stress Scale (JCQ without support component) | Past & present |
| DeSanto Iennaco et al. (2009) [138] | Cohort study, historical | North America (USA) | Hourly workers in heavy industry (aluminium manufactur) | 7,566 (T1: 95.3%, T2: 72%) | 18-64; 46.2 (±9.5), | 6% | Race, education, job grade, tenure) | Demand-Control-Model (externally rated) | Present |
| Dragano et al. (2007) [98] | Cross-sectional study (baseline of prospective cohort study), present | Europe (Germany) | Unselected working population | 1,811 (55.8%) | 45-65 | 40.9% | Education, occupational status, social integration index | JDC (16 items) from the JCQ  Effort-Reward-Imbalance (ERI) questionnaire, incl. overcommitment (OC) | Present |
| Du Prel et al. (2014) [99] | Cross-sectional study, present | Europe (Germany) | Socially insured employees, no self-employed, no civil servants | 6,339  (27.3%) | 52 & 46 | 53.1% | Education (regional unemployment, second level moderator) | ERI-Ratio, incl. OC | Present |
| Du Prel et al. (2014) [100] | Cross-sectional study, present | Europe (Germany) | Socially insured employees, no self-employed, no civil servants | 6,339  (27.3%) analysis sample 5906 | 52 & 46 | 50.8% | Education, negative affectivity,  overcommitment, age, no. of children <14 years | ERI-Ratio, incl. OC,  Work-Family-Conflict (WFC)-items of the COPSOQ | Present |
| Elsayed et al. (2017) [157] | Cross-sectional study, present | Africa (Egypt) | Psychiatric nurses | 70  (80.5%) | 20-40 | 62.9% | Marital status, education, years of experience | Devilliers,Carson & Leary stress scale | Unclear |
| Gayman et al. (2012) [139] | Cross-sectional study, present | North America (USA) | Probation/ Parole officers | 826 (67.8%) | 23-65; 39.23 (±9.23) | 53.9% | Race | Wheaton’s chronic stress scale (Organizational stress) | Present |
| Goodman et al. (2009) [140] | Cohort study, prospective | North America (USA) | Nursing, home care, child care, cashier, waitress, nurse, food preparation worker, office worker | 414 (not reported) | 27.93 (±5.42) | 100% | Partner status, educational level, race | Work environment scale/Work pressure subscale | Present |
| Gray-Stanley et al. (2010) [141] | Cross-sectional study, present | North America (USA) | Support professionals (for adults with ID) | 323 (47%) | ≥ 35 (53%) | 83% | Race, education, marital status | Self-constructed work stress instrument, work social support | Unclear |
| Hakanen et al. (2008) [115] | Longitudinal (panel) study | Europe (Finland) | Dentists | 3,035 (71%) | Not reported (‘repre-sentative for age range for dentists in Finnland’) | Not reported  (‘repre-sentative gender ratio for dentists in Finnland’) | - | Job Demand-Resource (JD-R) scales specific to dentists | Present |
| Hall et al. (2013) [153] | Cross-sectional study, present | Oceania  (Australia) | General working population without self-employed | 2,343 (30.7%) | 39.50 (±13.01) | 48% | Marital status (employment demographics: type of employment, type of organization) | JCQ; Psychosocial Safety Climate (PSC-12) | Present |
| Han et al. (2019) [40] | Cross-sectional study, present | China  (Asia) | Different (patients with benign breast disease) | 371 (93.5%) | 40.31 (±6.91) | Not reported | Education, marital status, monthly income, weekly working hours, occupation | ERI-23 item questionnaire | Present |
| He et al. (2018) [41] | Cross-sectional study, present | China  (Asia) | Medical doctors and nurses | 243 (not reported) | 18-62; 31.8 (±9.2) | 60.1% | Educational level | House & Rizzo Work Stress Scale (Chinese version) | Unclear |
| Hoshino et al. (2016) [55] | Cross-sectional study, present | Japan (Asia) | Not reported | 70 (not reported) | 30-60 | 100% | Working hours (job & housework) | National Institute of Occupational Safety and Health-Generic Job Stress Questionnaire (NIOSH-GJSQ) | Present |
| Hoven et al. (2015) [130] | Cohort study, prospective | Europe (11 countries) | Not reported | 2,798 (61%) | 50-64; 54.76 (±3.30) | 40.7% | Occupational class & status | Abbreviated versions of JDC & ERI questionnaires in combination | Unclear |
| Hybels et al. (2018) [142] | Cohort study, prospective | North America (USA) | Methodist clergy | 1172 (not reported) | 51.5 (±10.4) | 33% | Race, marital status | Clergy Occupational Distress Index (CODI) | Past |
| Inoue et al. (2009) [56] | Cross-sectional study, present | Asia (Japan) | Automotive manufacture workers | 20313 (85%) | 38.8 (±7.4) | 14.4% | Education, marital status, occupation | Interpersonal conflict, NIOSH-GJSQ | Unclear |
| Inoue et al. (2010) [57] | Cohort study, prospective | Asia (Japan) | Range of occupations at six manufacturing factories | 15,256  (85%) | 40.5 (±8.9) | 0% | Age, education, marital status, occupation, | Job overload; Job control; Job overload/control ratio; role ambiguity; role conflict; supervisor support; coworker support; job insecurity from  NIOSH-GJSQ (Japanese version),  Job insecurity from JCQ | Past |
| Inoue et al. (2016) [58] | Cross-sectional study, present | Japan (Asia) | Not reported | 231 (not reported) | 57.0(±8.8) | 14.3% |  | JCQ | Present |
| Jeon et al. (2018) [143] | Cross-sectional study, present | North America (USA) | Early childhood education teacher | 207 (45% for the active recruitement system, for the passive not reported) | 17-66; 38.2 (±13.1) | 98.1% | Ethnicity, education level | Job demands; Job resources; Child Care Worker Job Stress Inventory | Present |
| Jiang et al. (2018) [42] | Cross-sectional study, present | Asia (China) | Deep-sea fishermen | 1,068 (not reported) | 18-67; 38.1 (±10.8) | Not reported | Education level, marital status, religion | Heavy workloads, intense time pressures, latitude in decision-making, occupational risks, lack of support from co-workers, Mental Stressor Investigation Questionnaire  (MSIQ; Yu et al. 2014) | Present |
| Jolivet et al. (2010) [118] | Cohort study, prospective | Europe (France) | Registered nurses and nursing aids | 4,308 (91.4%) | registered nurses: 35.8 (±9.2); nursing aids: 40.5 (9.4) | 100% |  | ERI questionnaire incl. OC (French version); organizational work environment | Present |
| Katsuyama et al. (2008) [59] | Cross-sectional study, present | Asia (Japan) | Workers in a manufacturing company and a local hospital | 243 (80%) | 40.8 (±10.3) | 43% |  | Brief Job Stress Questionnaire (BJSQ) | Present |
| Keser et al. (2018) [90] | Cross-sectional study, present | Asia (Turkey) | Academics in University | 114 (67%) | Not reported (number in age group) | 48% | Educational level | ERI questionnaire (Turkish short version) | Present |
| Kikuchi et al. (2014) [60] | Cross-sectional study, present | Asia (Japan) | Nurses | 330 (57.6%) | 32.9 (±8.5) | 100% |  | Job quantitative overload; job control and support from supervisors and coworkers; BJSQ | Present |
| Kim et al . (2018) [80] | Cross-sectional study, past | Asia (South Korea) | Firefighters | 7,151 (not reported) | 39.7 (±9.0) | 9.3% | Length of work, rank | Attending traumatic events; Korean  Occupational Stress Scale short form (KOSS-SF) | Past |
| Kim et al. (2015) [81] | Cross-sectional study, present | Asia (South Korea) | Call  center employees | 150 (not reported) | 21-29 (7.4%); 30-39 (61.1%); 40-49 (25.9%); 50-59 (5.6%) | 89.5% | Position, monthly income | ERI questionnaire (Korean version) | Present |
| Kitaoka‐Higashiguchi et al. (2002) [61] | Cross-sectional study, present | Asia (Japan) | Intermediate managers in a manufacturing company | 687 (89.2%) | 35-60; 48.1 (±5.8) | Not stated |  | JCQ (Japanese version) | Present |
| Kolstad et al. (2010) [111] | Cross-sectional study, present | Europe (Denmark) | Various public sector employees | 4,291 (45%) | Not reported (number in age group) | ~80% | Educational level | COPSOQ | Present |
| Koreki et al. (2015) [62] | Cross-sectional study, present | Asia (Japan) | Psychiatrists | 154 (74.8%) | 34.3  (±5.2) | 28% | - | BJSQ (12-item abbreviated version); Visual  Analogue Scale | Present |
| LaMontagne et al. (2008) [154] | Cross-sectional study, retrospective | Oceania  **(**Australia) | Different occupations | 1,101 (66%) | Not reported | 52% | Occupational skill level; highest level of education completed | JDC-measures by Karasek | Past |
| Lee et al. (2012) [83] | Cross-sectional study, present | Asia (Korea) | Workers with migrant background and different occupations (59.4% service workers) | 170 (85%) | 55.53 (±8.23) | 69.4% | Marital status | Korean  Occupational Stress Scale (KOSS) (job demand, insufficient job control, interpersonal conflict) | Present |
| Lee et al. (2012) [82] | Cross-sectional study, present | Asia (Korea) | Nurses | 284 (not reported) | 24-38 (±2.82) | 100 | Marital status | KOSS-SF: job demand (8 items), job control (5 items), interpersonal conflict (4 items) | Past |
| Li J et al. (2013) [101] | Cohort study, prospective | Europe (Germany) | Junior physicians | 1,000 (62.1%) | 30.42 (±2.61) | 49.6 | Partnership, children (yes/no) | ERI questionnaire incl. OC | Present |
| Li Q et al. (2019) [43] | Cross-sectional study, present | Asia (China) | Migrant workers in different workplaces (e.g. retail stores, restaurants,  hotels, clubs, barbershops, clerk offices, factories,  and casinos) | 900 (not reported) | 31.3 (±7.86) | 42.4% | Marital status, educational  attainment, monthly income, the duration of work,  subjective socioeconomic status (SSES) | Occupational Stress Indicator (short form) | Present |
| Lin HS et al. (2010)  [91] | Cross-sectional study, present | Asia (Taiwan) | Psychiatric nurses | 141 (91.6%) | 34.99 (±8.74) | 100% | Marital status, number of children, living with other people, monthly income | Taiwanese Nurse Stress Checklist | Present |
| Lin TC et al. (2016)  [92] | Cross-sectional study, present | Asia (Taiwan) | Pediatric intensive care nurses | 144 (97.9%) | 35.72 (±7.11) | 100% | Marital status, number of children, religious beliefs, education level | Taiwanese Nurse Stress Checklist | Present |
| Liu et al. (2012)  [44] | Cross-sectional study, present | Asia (China) | Physicians | 998 (76%) | 35.92 (±7.62) | 55.1% | Marital status, education | ERI Ratio incl. OC | Present |
| Looseley et al. (2019)  [120] | Cross-sectional study, present | Europe (UK) | Physicians (Anaestetic trainees) | 397 (64%) | 26-47 | 48% | Marital status, parenthood status | Perceived Stress Scale (PSS) | Present |
| Lunau et al. (2013)  [161] | Longitudinal study with data from SHARE, ELSA, HRS, prospective | Europe& North America  (Different countries of both continents) | Different | 5,650; (SHARE:61.6%; ELSA: 82%; HRS: 87.8%) | 50–64 | not reported | Educational level, income | control dimension of the JDC-model; ERI (‘efforts’: 2 items & ‘rewards’: 5 items) | Past |
| Lunau et al. (2018)  [144] | Longitudinal study with data from the US Health & Retirement Study | North America (USA) | Different | 4,575, 898 of these  contributed twice (not reported) | ≥50; 60.9 | 51.8% | Educational level | JDC & ERI questionnaires | Past |
| Mackie et al. (2001)  [145] | Cross-sectional study, present | North America (USA) | Employees of human services residential care facility | 728 (81%) | 39 | 75% |  | Perceived Work Stress Scale | Past |
| Kuhnke-Wagner et al. (2010)  [102] | Cross-sectional study, present | Europe (Germany) | Executives | 154  (61.6%) | female: 42,8 (±7,6); male: 44,0 (±7,1) | 39% | Educational level | ERI questionnaire incl. OC | Present |
| Larisch  et al. (2003)  [103] | Cross-sectional study, present | Europe, (Germany) | Employees in transport companies (50 % bus drivers, 23 % working in repair and 23% in administration) | 316,  max. 1,000 informed -> 316 participated | ≥ 35; 44.6 (±7,5) | 16% | Educational level and household income | ERI questionnaire incl. OC | Present |
| Mäntyniemi et al. (2012)  [116] | Cohort study, prospective | Europe, (Finland) | Municipal services employees | 69,842  (68%) | 17-64; 44.3 | 76.2% | SES measure/estimate  (Occupational position) | JCQ | Present |
| McCleese et al. (2007)  [146] | Cross-sectional study, present | North America (USA) | Plateaued employees | 44 (not reported) | 24-60; 36.00 (±10.22) | 55% | Educational level, marital status | 4-item Perceived Stress Scale, Job content plateau stress assessed using the Cohen et al. (1983) measure | Present |
| Mezuk et al. (2011)  [147] | Cohort study, prospective | North America (USA) | N/A | 2,902  (2004: 68.3% and 2006: 74%) | ≥50; 60.42 (±7.14) | 53.5% | Educational level, marital status, race | Psychosocial Leave-Behind Questionnaire (ERI) | Present |
| Mino et al. (2006)  [63] | Interventional study, prospective | Asia (Japan) | Workers of a company manufacturing precision machinery | 58 (not reported) | 38 | 0% | Marital status | Uehata Stress Questionnaire, ERI questionnaire, | Present |
| Miyaki et al. (2012)  [64] | Cross-sectional study, present | Asia (Japan) | Employees of a nationwide manufacturing company | 2,266 (90.1%) | 21-65;  43.5 (±9.8) | 10.6% | Years of education, annual household income | JDC-S questionnaire, Kessler 6 (K6) scale | Present |
| Nakada et al. (2016)  [65] | Cross-sectional study, present | Asia (Japan) | Schoolteachers | 1,006 (66.5%) | 39.7 (±11.6) | 59.6% | Educational level, marital status | NIOSH-GJSQ | Present |
| Nitta et al. (2018)  [66] | Cross-sectional study, present | Asia (Japan) | School principals and vice-principals | 530 (principals: 58%; vice-principals 69%) | principals: 56.1 (±3.6); viceprincipals: 50.3 (± 4.7) | 20%prin-cipals, 14% viceprincipals | Educational level, marital status, | NIOSH-GJSQ | Present |
| Nourry et al. (2014) [119] | Cross-sectional study, present | Europe (France) | Nurse managers | 296  (67%) | 46.2 (±7.4) | 89% | Work seniority | ERI questionnaire incl. OC | Present |
| Park et al. (2008) [84] | Cross-sectional study, present | Asia (South Korea) | Employees of small- and medium-sized enterprises | 3,013  (69.9%) | 19-65 | 28.5% | Educational level, marital status, different Job characteristics | KOSS-SF | Present |
| Peter et al. (2016) [104] | Cohort study, prospective | Europe, Germany | Different | t_0_: 6,585 (27.3%), t_1_: 4,244 | 52 or 46 | 52 years: 45.6%, 46 years: 54.4% | Occupational position, education and training, status inconsistency (SSI) | ERI questionnaire incl. OC | Present |
| Proeschold-Bell et al. (2013) [148] | Cross-sectional (analysis of first wave data of a study over two waves), present | North America (USA) | Clergy | t_0:_ 1,726 (95%); t_1:_ 2,008 | 40-60 | 25.3% | Educational level, marital status, race | Clergy  Occupational Distress Inventory | Present |
| Pulkki  -Raback (2015) [117] | Cohort study, prospective | Europe (Finland) | Different | 1,546 (91.8 %) | 37.5 | 60.7% | - | Demands (3 items from the Finish Occupational Stress Questionnaire)  Control (9 items from the JCQ) | Past |
| Raskin et al. (2014) [129] | Cross-sectional study, present | Europe (Ukraine) | Orphanage caregivers  and preschool teachers | 120  (~50 %) | 42.17 (±1.14) | 100% | Marital status, number of own children, salary, husbands’ salary, education, husband’s education | Lack of job resources and control measured by the Child Care Worker Job Stress Inventory (CCW-JSI) | Present |
| Rayens et al. (2013) [149] | Cross-sectional study, present | North America (USA) | Farmers | 988 (40 %) | >50; 64.1 (±7.9) | 50% | Minority, marital status, annual household income | PSS (shortened version) | Past |
| Rösler et al. (2008) [105] | Cross-sectional study, present | Europe  (Germany) | Different | 265 (96%) | 20-64; 43 (±10) | 67.5% | - | JDC-S (FIT, SALSA) & ERI-questionnaires | Present |
| Rugulies et al. (2006) [112] | Cohort study, prospective | Europe (Denmark) | Different | t1:10,702, t2: 4,133  (80%) | 39 (±11) | 48.5% | Family status, school education, occupational position | Quantitative demands, influence at work, possibilities for  development, social support from supervisors, social support  from coworkers, job insecurity. |  |
| Saijo et al. (2014) [67] | Cross-sectional study, present | Asia (Japan) | Physicians | 494 (19.4%) | 24 - >50 | 19.9% | Marital status | BJSQ | Present |
| Saijo et al. (2014) [68] | Cross-sectional study, present | Asia (Japan) | Civil servants | 2,121 (89.4%) | 19-60 | 31.2% | Marital status, employment grade | BJSQ |  |
| Saijo et al. (2007) [69] | Cross-sectional study, present | Asia (Japan) | Fire fighters | 1,672 (98%) | 18-60 | 2.8% | Marital status, job class | NIOSH-GJSQ (Japanese version) | Present |
| Saijo et al. (2016) [70] | Cross-sectional study, present | Asia (Japan) | Nurses | 1,063 (90.1%) | <29 - >50 | 93.3% | Education, marital status, income, job rank | BJSQ | present |
| Sakata et al. (2008) [71] | Cross-sectional study, present | Asia (Japan) | Medical residents | 196 (86.3%) | 24-44; 37.3 (±2.9) | 24% | Year of residency, single or cohabitation | ERI- questionnaire (Japanese version) incl. OC | Present |
| Sandström et al. (2012) [123] | Clinical study | Europe (Sweden) | Patients with depression in comparison with those being long-term absent due to work stress and healthy subjects | 30  (100%) | 37.3 (±4.1) | 100% | - | PSQ | Present |
| Santa Maria et al. (2017) [106] | Cross-sectional study, present | Europe (Germany) | Police officers | 843 (50.7%) | 40.9 (±9.0) | 27.8% | Length of service | JD-R questionnaire | Present |
| Shang et al. (2015) [45] | Cross-sectional study, present | Asia  (China) | Different | 2,457 (85%) | 39.86 (±4.21) | 49.17% | Marital status,  subjective socioeconomic status | ERI-Ratio (Chinese short version) | Present |
| Shen et al. (2014) [46] | Cross-sectional study, present | Asia (China) | University teachers | 1,210 (80.7 %) | 39.15; (±8.02) | 57.6% | Marital status, education, professional position | ERI-Ratio incl. OC (Chinese version) | Present |
| Shepard-Binigan et al. (2016) [150] | Longitudinal study, prospective | North America (USA) | Women with young children | 570 (41.7%) | 29.6 (±4.7) | 100% | Number of children in household, total family income, race/ethnicity, education, partner employment status | Job Role Quality Scale | Present |
| Siegrist et al. (2012) [162] | Cohort studies, prospective (SHARE; ELSA; HRS; JSTAR) | Europe, North America, Asia  (15 European countries, USA, Japan) | N/A | 14,236 cross-sectionally/ 6,657 longitudinally (not reported) | 50-64 | Europe 47.2%; USA 55.3%; Japan 40% | Educational level | Low control, ERI questionnaire | Present & Past |
| Simmons et al. (2009) [151] | Cross-sectional study, present | North America (USA) | Employees in professional, administrative, executive, managerial jobs and service occupations | 2,584 (52%) | ≥18 (58% between 26 & 49) | 48.6% | Ethnicity, educational level, marital status, children (yes/no), poverty, urbanicity level, urbanicity | Seven variables that reflect current trends in research on the psychosocial work environment (Physical demands, psychological demands, decision latitude, workplace flexibility, supervisor support for work and family, coworker support, job insecurity) | Present |
| Smith et al. (2012) [152] | Cohort study, prospective | North America (Canada) | N/A | 3,735 (69%) | 25-60 | 45.7% | Marital status, number of children < 12 y in the household, level of education, | JCQ (abbreviated version) incl. social support | Present |
| Stansfeld et al. (2012) [121] | Cohort study, prospective | Europe (UK) | Civil  servants | 3,942 (73%) | 45-64 | 26.0% | Marital status, employment grade, education level | Adapted version of JCQ (job strain incl. support) | Present |
| Steinhardt et al. (2011] [163] | Cross-sectional study, present | North America (USA/Texas) | Public school teachers | 267 (26%) | 23-68; 45 | 75.0% | Ethnicity, education level | Modified version of the Teacher Stress Inventory (Fimian, 1984), | Past and present |
| Takaki et al. (2010) [72] | Cross-sectional study, present | Asia (Japan) | Employees in manufacturing companies, healthcare or welfare institutions | 1,646-2,062  (62.5-78.2%) | Men: 41.6 (±13.5); women 39.4 (±12.1) | 62.6% | Occupational position, annual household income | JCQ (Japanese version) incl. social support, workplace bullying | Present |
| Tatsuse et al. (2019) [73] | Cohort study, prospective | Asia (Japan) | Civil servants | 992 (80.6%) | 19-65: 43.1 (±12.8) | 44.7% | Marital status | JDC-S questionnaire | Present |
| Theorell et al. (2014) [124] | Cohort study, prospective | Europe (Sweden) | N/A | 6,177 (61%) | 16-64; Men: 51.07 (±11.33); women: 49.93 (±11.53) | 56% | Income, marital status | JDC- questionnaire (Schwedish shortened version) | Present |
| Tomioka et al. (2011) [74] | Cross-sectional study, present | Asia (Japan) | Physicians | 706 (42%) | 37.4 (±6.7) | 24.4% | Years of clinical experience, speciality | ERI (23 item version) incl OC, social support from NIOSH-GJSQ | Present |
| Tsuboi et al. (2006) [75] | Cross-sectional study, present | Asia (Japan) | Nurses | 33 (55%) | Low job stress group - 30.2 (±9.63), high job stress group 26.2 (±5.41) | 100% | No | BJSQ | Past |
| Tsutsumi et al. (2001) [76] | Cross-sectional study, present | Asia (Japan) | Manual and non-manual employees of direct assembly line and indirect supportive tasks (the latter target to downsizing) | 190 (89%) | 20-59; 36.5 (±9.0) | 47% | Occupational status | JDC (Japanese version) & ERI (incl OC)- questionnaires | Present |
| Tsutsumi et al. (2011) [77] | Cross-sectional study, present | Asia (Japan) | Private practice physicians | 1,103 (38%) | 58 (±11) | 7.3% | - | ERI questionnaire (short version without OC) | Present |
| Vearing and Mak (2007) [155] | Cross-sectional study, present | Oceania (Australia) | Employees from two retail organisations and employees from a public service organization | 224 (retail sector:28%; public service sector: 46%) | <20 - 64 | 51.8% | Education, type of work | ERI questionnaire incl. OC, Social Support Scale |  |
| Wallace (2006) [133] | Cross-sectional study, present | North America (Kanada) | Lawyers | 1,201 (31%) | 42 | 30% | Marital status, preschool children at home, adequate income, partner's employment status | JDC-S questionnaire | Present |
| Wang C et al. (2016) [47] | Cross-sectional study, present | Asia (China) | Electronic Manufacturing Service Employees | 1,618 (89.9%) | 28.84 (±6.37) | 48.4% | Marital status, education, occupational position | Job burden capital questionnaire (JCQ+ERI incl. OC) | Present |
| Wang J et al. (2010) [134] | Cohort study, prospective | North America (Kanada) | No specific working population | 6,008 (  83.60%) | 39.80 (±0.21) | 44.2% | Marital status, education, household income | JCQ (short version) | Present |
| Wang J et al. (2009) [135] | Cohort study, prospective | North America (Kanada) | No specific working population | 4,866 (83.6%) | 36.16 | 45.9% | Marital status, single parent family structure, education, annual family income | JCQ (short version) | Present |
| Wang J et al. (2011) [136] | Cross-sectional study, present | North America (Kanada) | No specific working population | 4,302 (44%) | 25-65 | 46.3% | Marital status, education, personal annual income, occupational position | ERI, JCQ, WFC | Present |
| Wang LJ et al. (2011) [93] | Cross-sectional study, present | Asia (Taiwan) | Physicians (in comparison to other occupations) | 1,643 (86.3%) | <30 - >51 | 15.2% | Marital status | JCQ | Present |
| Wang SM et al. (2015) [94] | Cross-sectional study, present | Asia (Taiwan) | Psychiatric nurses | 154 (81%) | 21-53; 32 (±6.77) | 92.9% | Marital status, religion, education | Nurse Stress Checklist: single items (personal responses, work concerns, competency, incompleteness of personal arrangement) | Present |
| Wege et al. (2018)  [107] | Other longitudinal study, prospective | Europe (Germany) | No specific occupational group | 6,693 (80.7%) | 44.11 (±11.14) | 49.4% | marital status, education, income | ERI questionnaire (short form) incl. OC | Present |
| Wu et al. (2011) [48] | Cross-sectional study, present | Asia (China) | Nurses | 1,986 (78.4%) | 34.5 (±9.6) | 100% | Marital status,  education | Chinese version of Occupational Stress Inventory-revised edition | Past time |
| Yoon SL, Kim JH (2013) [85] | Cross-sectional study, present | Asia (Korea) | Nurses | 441 (86.7%) | 28.2 (±5.4) | 100% | Monthly salary, marital status, occupational position | KOSS-SF | Present |
| Yoshizawa K et al. (2016) [78] | Cross-sectional study, present | Asia (Japan) | Psychiatric nurses | 238 (74.8%) | 45.8 (±12.9) | 72.3% | Marital status, with / no children | NIOSH-GJSQ | Present |
| Li W et al. (2019) [49] | Cross-sectional Secondary data analysis | Asia (China) | Migrant workers, any employment | 1,434 (not reported) | 15-71; 36.47 (±11.91) | 54.3% | Marital status, education | 3-item self-constructed scale asking how often (1) work is exhausting, (2) work is always stressful & (3) losing interest in job more and more | Present |
| Kim et al. (2020) [137] | Cross-sectional study, present | North America (Canada) | Not specified | 1,288 (not reported) | 30-70; 49.72 (±8.96) | 48,7% | Marital status, years since immigration, pre-immigration education, Canadian education, household income | Emotional demand scale; Occupational resources were measured by Job autonomy scale (4 items: task control, decision making responsibility, decision freedom & work schedule flexibility); Job Satisfaction Scale of the Michigan Organizational Assessment Scale; Job security (two items) | Present |
| Sun et al. (2020) [50] | Cross-sectional study, present | Asia (China) | Any | 5,751 (not reported) | ≥ 50; urban: 56.01 (±5.13); rural: 58.79 (±6.69); migrant: 54.64 (±4.32) | Urban: 29.5%; rural: 43.4%  -rural; migrant 37.2- | Educational level, marital status, self-rated class. urban/rural status, migrant status | work stress, family debt, family relationships, neighborhood cohesion, environmental threats;  3-item scale developed by Cai and Jian (2017) | Present |
| Åhlin et al. (2018)  [125] | Cohort study, prospective | Europe (Sweden) | Any | 7,949 (57-65%) | Wave 2: <35 (10%), 35-49 (33.8%), >= 50 (56.2%) | Wave 2: 56.1% | No of children at home, civil status (married, cohabiting) | JDC questionnaire (Swedish shortened version) | Present |
| Magnusson Hanson et al. (2014)  [126] | Cohort study, prospective | Europe (Sweden) | Any | 2,017 | 16-64 | 55.2 | Education, marital status | Demands and Support items of the JDC-S  questionnaire (Swedish version) | Present |
| Magnusson Hanson et al. (2009) [127] | Cross-sectional study, retrospective | Europe (Sweden) | Any | 5,985 (65%) | 16-64 | 54.5% | Education, job position, marital status, industrial sector, employment status | Demands, decision authority, social support, and conflicts  at work (based on Swedish Work Environment Survey) | Present |
| Schramm et al (2020) [108] | Interventional study, prospective | Europe (Germany) | Any | 28 (100) | 47 (interventional) vs. 52 (control) | 78.6% | Married or cohabiting; academic degree; Size of business employer | ERI-questionnaire (short version) incl. OC | Present |
| Wu et al. (2021) [51] | Cross-sectional study, retrospective | Asia (Hainan Province China) | Fisherman | 229 (30.2%) | 16-65; 35.16 (±10.96) | 0% | Education, marital status,  religion | MSIQ (developed to assess the working stress of naval sailors) | Past |
| Åhlin et al. (2021)  [131] | Cohort study, historical | Europe (Sweden & Denmark) | N/A | 3,707 (Panel 1: 45%; Panel 2: 47%) from Sweden (SLOSH); 5,496 (53.4%) from Denmark (WEHD) | 16-64 (SLOSH), 18-64 (WEHD) | 55.2% (SLOSH), 52.2% (WEHD) | Marital status | Job demands items (work pace, conflicting demands, enough time) of the JDC questionnaire | Past |
| Jung et al. (2020) [86] | Cross-sectional study, retrospective | Asia (Korea) | Nurses | 291 (72,8%) | 20-49 | 94,2% | Marital status | KOSS-SF | Past |
| Beschoner et al. (2021) [109] | Cross-sectional study (2 times), prospective | Europe (Germany) | Psychiatrists | Complete information was available from N = 997 in 2006 (89.2%) and N = 800 in 2016 (90.6%) (51%) | 44.40 (±8.55) | ~50% | Marital status | Working hours (average weekly working hours and weekend)  ERI questionnaire incl. OC | Present |
| Yang et al. (2020) [52] | Cross-sectional study, present | Asia (China, Hong Kong) | Any | 1,352 (71.8%) | 18-60 | 0% | Current marital/cohabitation status, educational level, | Workaholism [7-item Bergen Work Addiction Scale]  Work-life balance stress  Self-constructed question: ‘To what extent you perceive stress to keep your work-life balance?’ | Present |
| Weigl et al. (2021) [110] | Cross-sectional study, present | Europe (Germany) | Geriatric and registered nurses & some working in other health care professions | 370 | 18-63; 35.9 (±11.4) | 85% | Educational level, examination status | Trier Inventory of Chronic Stress | Past |
| Mohamed AF et al. (2022) [88] | Interventional study (RCT) | Asia (Malaysia) | Manufactory workers | 88 | 18-65 | 100% | Marital status, educational level, years of employment, job position, monthly salary, race, religion | Self-perceived stress (DASS-21 stress scale); JCQ | Present |
| Mayerl et al. (2020) [132] | Cohort study, prospective | Europe (9 countries: AUT, BEL, CHE, DEU, DNK, ESP, FRA, ITA, SWE) | Different | 5,778 (not reported) | 50-85; 55.0 (±4.3) | 46% | Educational level | ERI questionnaire | Present |
| Yong et al. (2020) [53] | Cross-sectional study, present | Asia (China, Province Xinjiang) | Coal miners | 1,344 (95.3%) | 18-60; 42.2 (±8.6) | 12.1% | Working years,  level of education, annual income | ERI questionnaire (Chinese version) incl. OC | Present |
| Hsieh et al. (2021) [95] | Cross-sectional study, present | Asia (Taiwan) | Psychiatric ward nurses | 248 (95,4%) | 20–52; 32.98 (±8.25) | 100% | Marital status, religious belief, college graduation | BSS] | Present |
| Mohamed MY et al. (2023) [158] | Cross-sectional study, present | Africa (Egypt) | Medical residents | 220  (100%) | 26.97(±1.01) | 56.4% | Educational level | Sociodemographic & -economic stressors [The Hospital Consultants Job Stress and Satisfaction Questionnaire (HCJSSQ)] | Past time |
| Kploanyi et al. (2020) [159] | Cross-sectional study, present | Africa (Ghana) | Employess of a telecommunication company | 235 (96%) | 20-49; 30.8 (±6.9) | 47.2% | Marital status | NIOSH-GJSQ | Past time |
| Almroth et al. (2022) [128] | Register-based study, longitudinal | Europe (Sweden) | Any | 3,011,545 | 30-60 | 50.1% | Education, birth country, civil status, number of children, | JDC measured by job exposure matrix (Fredlund, Hallqvist, & Diderichsen, 2000) based on the Swedish Work Environment Surveys (1997–2013) | Present |
| Kim et al. (2020) [87] | Cohort study, prospective | Asia (Korea) | Any | 63,959 (87.59%) | ≥18; men: 37.75 women: 33,78 | 21.2% | Marital status, education, income, | KOSS-SF | Present |

^a^if only age groups are given an estimate of the mean value is calculated where possible

Abr.: ASSET = A Shortened Stress Evaluation Tool; BJSQ = Brief Job Stress Questionnaire; CCW-JSI = Child Care Worker Job Stress Inventory; CODI = Clergy Occupational Distress Index; ERI = Effort-Reward Imbalance; JCQ = Job Content Questionnaire; JDC-S = Job Demand Control-Support; JD-R = Job Demand-Resource; KOSS(-SF) = Korean Occupational Stress Scale (-Short Form); MSIQ = Mental Stressor Investigation Questionnaire; NIOSH-GJSQ = National Institute of Occupational Safety and Health Generic Job Questionnaire; OC = Overcommitment; PHQ-9=Patient Health Questionnaire-9; PSQ = Perceived Stress Questionnaire; PSS = Perceived Stress Scale ; WFC = Work-Family-Conflict

**Supplementary Table S2B Study characteristics, Scoping review on the relationsship between work-related stress and depression (six continents, 1999-2022) (continued)**

| **Author (year) + [Ref.]** | **Outcome: Measure of depression/ depressiveness**  **[scale] OR/AND doctors diagnosis** | **Categorised or continuous measure of outcome** | **Effect size ´(KI OR p-value)** | **Factors for which effect sizes adjusted for^b^** | **Key conclusions** | **Remarks** |
| --- | --- | --- | --- | --- | --- | --- |
| Ahola et al. (2007)  [113] | Beck Depression Inventory, short form (BDI-V) | Categorised | OR = 3.4 (95%-KI: 2.0-5.7) | Sex, age and marital status at baseline. | Association for high job strain to depression – 3.4 (2.0-5.7), but it disappeared when adjusted for burnout. Job strain predicted depression of those who had no born out at baseline - the adjusted probability for depr. was 7.5 (3.5-15.9) for each 1-point increase of job strain score. There is a reciprocal relationship between burnout and depressive symptoms. |  |
| Ahola et al. (2006)  [114] | Beck Depression Inventory (original) (BDI) | Categorised | Depressive symptoms: OR = 3.8 (95% CI: 2.8 –5.1])  Depressive disorders: OR = 1.7 (95% CI: 1.1–2.6) | Sex, age | The risk for depressive symptoms and for depressive disorders of high strain was reduced by 69% or more after adjusting for burnout. |  |
| Armon et al. (2010)  [54] | Personal Health Questionnaire (PHQ) for depressive symptomatology,  PRIME-MD for depression | Continuous (depressive symptoms), Categorized (depression) | Multiple regression analysis in T1–T2 showed signiﬁcant association between work load and depressive symptoms as well as inverse association between social support and job control on depressive symptoms in men (not in women) | Depressive symptoms at baseline, neuroticism, age, education, follow-up duration | The study showed the bi-directional association between work characteristics and depressive symptoms in which they mutually influence each other, at least for the mean levels (β=.12) and in T1–T3 (β=.11) |  |
| Bailey et al. (2015)  [156] | PHQ-9 | Continuous | Job strain (T1) was positively associated with depressive symptoms (T2): ß = 0.08, B = 0.61, SE 0.21 (p *<* 0.01),  F(5, 1075) = 70.32 (*p* <0.001) | Depressive symptoms (T1), | Job strain predicts depressive symptoms | The authors mainly concentrate on psycho/social climate (PSC) benchmarking for practical use and to diminish mental problems (job stress, depression). |
| Balog et al. (2003) [122] | Perlin 9-item questionnaire (validated by BDI, internal consistency by Cronbach alpha 85%) | Continuous | Neither in the study (p = 0.09) nor in the control group (p = 0.06) were work stress significantly associated with depression in the multiple adjusted model | Age, educational level, status of menopause, BMI,  smoking, sedentary lifestyle, Killip classification for patients | Marital  stress but not work stress is independently related to depressive  symptoms in women. | Study investigated women with CHD and healthy women as control group |
| Batalla et al. (2018) [89] | BDI-II | Categorized | Correlation between work stress and depression: r = 0.32 (p < 0.05) |  | Although higher spirituality leads to lower depression scores, severe occupational stress as a moderator may curtail the protective influence of spirituality. | Aim: to determine the influence of spirituality to depression and to determine the moderating effect of occupational stress among Registered Nurses |
| Bernburg et al. (2016) [97] | ICD-10 depr symptoms (0-4) | Continuous | ß = 0.28 (p= 0.01) | Age, gender, marital status, presence of children, medical specialty, years of experience | Occupational distress was positively associated to depressive symptoms, but negatively with work ability. Significant differences between medical specialties in working conditions,  occupational distress, depressive symptoms and work ability. Quantitative demands (ß = 0.26) and influence at work (ß = -0.21) significantly associated to depressive symptoms. | This study aimed to analyze and compare  differences in occupational stress, depressive  symptoms, work ability and working environment  among residents working in various medical specialties. |
| Chen et al. (2009) [39] | Center for Epidemiological Survey-Depression Scale (CES-D) (Chinese version) | Continuous | ß = 0.16 (p < 0.01) | Education level,  organizational types  age,  gender,  different interaction terms | Employees who were exposed to higher level of job stressors were more likely to report depressive symptoms. The findings also generally confirm the direct and moderating effects of informal social support (Subjective informal social support and passivity were found to have direct effect on employees’ depression. Further, objective informal social support and distancing buffered the negative effect of job stressors on depression) | The primary aims of this study were to further  our understanding of the influence of job stressors  on employee mental health (depression) and to  examine stress moderators that might mitigate  this relationship. |
| Cho et al. (2008) [79] | CES-D | Categorised | Work stress subscales – depression:  inadequate social support OR = 1.58 (95%-CI: 1.52-1.66); discomfort in occupational climate OR = 1.25 (95%-CI: 1.18-1.30); lack of reward lack of reward OR = 1.14 (95%-CI: 1.09-1.19); insufficient job control OR = 1.08 (95%-CI: 1.03-1.14); high job demand OR = 1.06 (95%-CI: 1.03-1.09); job insecurity OR = 1.05 (95%-CI: 1.01-1.09) (Tab 3). | Gender, age, marital  status,  educational level, duration of work, industrial classification, all other KOSS factors | Inadequate social  support and discomfort  in occupational climate  were more important risk factors for depression than organizational  injustice, job demand and job control |  |
| Clays et al. (2007) [96] | CES-D (shorter Iowa form) | Categorized | High job strain OR = 1.74 (95%-CI: 1.00–3.01), isolated strain OR = 2.53 (95%-CI: 1.32–4.86), high job demands OR = 1.18 (95%-CI: 0.72–1.94), low decision lattitude OR = 1.90 (95%-CI: 1.08–3.33) increased risk of depression for women, only. Repeated high job strain (5,2%) had the highest incidence of depression symptoms for men OR = 3.31 (95%-CI: 1.67–6.56) and women OR = 3.40 (95%-CI: 1.45–7.94). Repeated isolated strain (1,9%) had an association to depressive symptoms only for men OR = 5.80 (95%-CI: 2.12–15.85) | age, educational level, social network, satisfaction with private life, locus of control, score for depression symptoms at T1. | This study confirms that job stress is a risk factor for developing symptoms of depression. Stronger associations were found for women. The impact of high job strain among both men and women was more harmful when there was repeated exposure. | The first objective was to describe prospectively the impact of job stress according to the demand–control–support model on the development of depression symptoms. The second objective was to relate repeated exposure to job strain with depression symptoms. |
| Da Silva Gherardi-Donato et al. (2015) [160] | Only 1Q interview. Has any doctor or health professional ever said that you have depression? | Categorised | High vs low job stress and depression prevalence: OR = 2.03 (p = 0.048) | Gender, early stress, age group, time of employment in the institution | Depressive symptoms were strongly associated with high stress levels among nursing assistants and nursing technicians. | Limitations caused by diagnostic and memory biases in reporting of depression at some point in life, and “Has any doctor or health professional ever said that you have depression?”.  The memory biases are established, once the outcome was measured  retrospectively, which can also lead to underestimation of the magnitude of depression. |
| DeSanto Iennaco et al. (2009) [138] | Doctors diagnosis (ICD 9), available from health insurance claims | Categorised | High vs low demand: OR = 1.39 (95%-CI: 1.04-1.86)  Control only significant in the unadjusted model | Age, gender, race, education, job grade, tenure, smoking status, body mass index,  cholesterol level) | This study finds that heavy industrial workers in jobs of high demand and moderate control have greater risk of depression diagnosis claims, but with full adjustment including location, these effects lose significance. Low control jobs were not associated with increased risk of depression diagnosis. | This study examined whether externally rated job demand and control were associated with depression  diagnosis claims in a heavy industrial cohort |
| Dragano et al. (2007) [98] | CES-D | Categorised | Control: OR = 1.87, (95%-CI: 1.28-2.73); ERI: OR = 3.36 (95%-CI: 2.11-5.10); overcommitment: OR = 3.94 (95%-CI: 2.66-5.83); interaction between control & overcommitment: OR = 2.14 (95%-CI:1.12-4.06) | Age, sex, education, occupational status, working hours, social integration, chronic disease, smoking, physical inactivity, other stress measures | Components of two common stress models (ERI, overcommitment, control) and their interaction (Control overcommitment) are associated with depressive symptoms in an unselected working population. |  |
| Du Prel et al. (2014) [99] | BDI-V | Continuous | Association between ERI-ratio and BDI-V total: ß = 6.063 (p < 0.001); ERI-ratio and social isolation as significant mediators identified | Age, gender, negative affectivity, overcommitment | findings point out that work-related  stress and social isolation play an intermediary role  between education and depressive symptoms in middle aged  employees. | Study question. Are effort-reward imbalance and social isolation mediating the association between education and depressiveness? B |
| Du Prel et al. (2014) [100] | BDI-V | Continuous | Total effect of ERI to depressive symptoms: Women (full time): ß = 6.61 (p < 0.001)  Women (part time): ß = 4.87 (p < 0.001)  Men (full time): ß = 8.02 (p < 0.001) | Education, negative affectivity,  overcommitment, age, no. of children < 14 years | Altogether this investigation provides cross-sectional evidence for a mediating effect of WFC in the association between work-related stress and depressive symptoms in full- and part-time employed middle-aged women and men in full-time positions. | Due to low number of male employees in part-time positions the mediation effect of WFC between ERI and depressive symptoms cannot be clarified |
| Elsayed et al. (2017) [157] | BDI-II | Continuous | Work stress & depressiveness: r = 0.71 (p = 0.01) | - | The results revealed that psychiatric nurses had moderate levels of work-related stress and depression, | No |
| Gayman et al. (2012) [139] | CES-D | Continuous | Work stress b = 0.18, SE: 0.04 (p < 0.001); role conflict b = 0.16, SE: 0.04 (p < 0.001) | Gender, age, race, job tenure | Depressive symptoms predicted by work stress, role conflict and burnout; burnout is a mediator |  |
| Goodman et al. (2009) [140] | Brief Symptom Inventory-18 (BSI-18); CES-D | Continuous | Work pressure: r = 0.15 (p < 0.01), negative work family spill over: r = 0.26 (p < 0.001) |  | Less flexible work environment & greater work pressure predicted higher depressiveness; mediated by negative work-family spillover |  |
| Gray-Stanley et al. (2010) [141] | CES-D | Continuous | Work stress b = 0.08, SE: 0.02 (p < 0.001); Work support b = -0.08, SE: 0.03 (p < 0.01) | Age, gender, race, education, marital status + living arrangement, caregiving responsibilities at home, supervisory status, tenure in organization | Work stress positively associated with depression, work resources negatively |  |
| Hakanen et al. (2008) [115] | BDI | Continuous | Job demands ß=0.24 (p< 0.001) | Age, gender; home resources & home demands | Job demands predicted burnout which predicted depression over time; job resources weak negative impact on burnout; home resources or demands did not influence health and wellbeing; job demands also direct effect on depression |  |
| Hall et al. (2013) [153] | PHQ-9 | Continuous | SPC (standardized path coefficient): job demands: 0.32***; PSC-12: -0,13***; job demands x PSC: -0,07**  Control was negatively associated with depression (0.24**) when controlled for demands |  | Psychological safety climate moderated effects of job demands on depression and further moderated effects of depression on positive organizational behavior |  |
| Han et al. (2019) [40] | CES-D | Continuous | ERI-Ratio to depressive symptoms: ß = 0.274 (95%-CI: 0.271-0.279)***, Psychological capital: ß = -0.339 (95%-CI: 0.331-0.345)*** | Psychological capital, age | Depressive symptoms positively correlated with ERI-Ratio, PsyCap associated with ERR mediated depressive symptoms |  |
| He et al. (2018) [41] | Zung Self-rating Depression Scale (SDS) | Continuous | Work stress: p < 0.001 | Gender, age, education, Val66Met, Stress * Val66Met | Significant correlation job stress depressiveness; effect of BDNFVal66Met genotype on depressive symptoms |  |
| Hoshino et al. (2016) [55] | CES-D | Categorized | OR (95%-CI): Interpersonal conflict (NIOSH GJSQ): 1.592 (1.208-2.099)**; quantitative workload (NIOSH-HK): 0.665 (0.477-0.927)*; variance in workload (NIOSH-HK): 4.077 (1.467-11.33)**; underutilization of abilities (NIOSH-HK): 2.061 (1.150-3.694)*; social support (NIOSH-HK): 0.846 (0.764-0.938)** | Besides different stress measures none | More factors in housework than in paid work associated with depression; in paid work interpersonal conflict was significant |  |
| Hoven et al. (2015) [130] | EURO-D depression scale | Continuous | Unstandardised path coefficient (95%-CI): ERI: 0.098 (0.031,0.165); low control: 0.068 (-0.005, 0.140) | County-affiliation, sex, age | ERI and low control mediate impact of occupational position (parametrized by occupational class and status) on depression |  |
| Hybels et al. (2018) [142] | PHQ-8 | Categorized | Mild depr. symptoms: OR=1.15 (95%-CI:1.09-1.21)***, severe depr. symptoms: OR 1.38 (95%-CI:1.29-1.49)*** | Age, gender, race, marital status | Higher levels of occupational distress, higher odds of more severe depressive symptoms |  |
| Inoue et al. (2009) [56] | CES-D | Categorized | OR_high vs low SES_ (95%-CI) of interpersonal conflict in the worksite for depression:  Male: 4.88 (4.04-5.90)  Female: 3.28 (1.89-5.69) | Age, marital status, overtime in the past month, chronic physical conditions, smoking status, drinking status, physical activity, supervisor support and coworker support. | Males of higher SES are more vulnerable to interpersonal conflict at work in terms of developing depression |  |
| Inoue et al. (2010) [57] | CES-D | Continuous | HR (95%-CI): Job control_high vs low_ 0.28 (0.11 - 0.71)**; job strain _high vs low_ 2.00 (0.92-4.39); Role ambiquity_high vs low_ 3.49 (1.43 -8.49)**; Role ambiquity_moderate vs low_ 2.66 (1.10 to 6.45* | Age, education, marital status, occupation and chronic physical conditions,  depressive symptoms,  neuroticism | The present study provided evidence for low job control, and moderate and high role ambiguity increasing a risk of long-term sick leave due to depressive disorders. High job strain was no longer significant after adjusting for depressive symptoms and neuroticism at baseline. |  |
| Inoue et al. (2016) [58] | SDS | Continuous | Job strain index to depressive symptoms: b =15.66, SE: 3.38*** | Age, sex, coronary artery disease, diabetes, hypertension, hyperlipidemia and current smoking status | Occupational stress expressed as the job strain index was strongly associated with depression. | Results can be influenced by a combination of different forms of bias |
| Jeon et al. (2018) [143] | RAND health depression screening tool | Continuous | Standardized path coefficient: 0.25*** | Years of education, major in ECE, years of experience teaching in ECE settings | The current study found that ECE teachers who had depressive symptoms tended to have lower professional motivation and more job-related stress. |  |
| Jiang et al. (2018) [42] | CES-D, Chinese version | Continuous | Direkt effect: Standardized path coefficient ß = 0.577***;  Other part mediated over nicotine dependence (ß = 0.054***) |  | Structural equation modeling (SEM) analyses showed that work stress and nicotine dependence were independent predictors of depressive symptoms. The relationship between work stress and depressive symptoms was found to be partially mediated by nicotine dependence and be moderated by cognitive reappraisal. | Results may have been influenced by selection bias |
| Jolivet et al. (2010) [118] | CES-D | Continuous | Multilevel analysis (fixed effects): ERI_high vs low_: b = 5.08***; Overcommitment _high vs low_:b = 4.16***; bad relationship between workers: b = 0.26**; Moreover ERI seems to be a mediator between ‘low level of communication in the work unit’ as well as ‘staffing inadequacy to perform work’ and depressiveness | Age, profession, specialty of the work unit, work schedule | Our study and our theoretical model allowed us to identify and quantify organizational factors that are associated with mental health either directly or indirectly through perceived ERI |  |
| Katsuyama et al. (2008) [59] | Depressed mood measured by BJSQ | Continuous | Not presented |  | Depressed mood shown in the present study did not present as a disease, but as a response to job stress. Job stress may elicit biological responses that contribute to depressed mood in relation to 5HTT polymorphisms, and social support may reduce depressed mood irrespective of 5HTT polymorphisms. |  |
| Keser et al. (2018) [90] | CES-D (Turkish version) | Categorized | OR = 3.80 (95%-CI: 1.67; 8.65) per SD of ERI-Ratio; OR = 7.39 (95%-CI: 2.15; 25.42) for high work stress group (ERI-Ratio > 1) | Age, sex, education, tenure, and occupational position | The findings indicated consistent associations of the single scales, namely “Effort” and “Reward,” and in particular of the theoretically important summary measure of the ERI-Ratio, with elevated risks of depressive symptoms in this sample. | Method of recruitment not stated |
| Kikuchi et al. (2014) [60] | Screening scale with items developed by Fukisawa et al. (2005) | Continuous | ß = 0.20, SE: 0.24 (p < 0.05) | Temperament, overtime hours | Depressive and cyclothymic temperament types significantly, directly and indirectly influenced depressive symptoms via job stress among female nurses in the general hospital |  |
| Kim et al . (2018) [80] | PHQ-9 | Continuous | Perceived job stress and depressive symptoms: b = 0.006 (95%-CI: 0.005-0.007)*** | Age, gender, work length, current job, resilience, traumatic events, PTSD symptoms | The results from this study suggest that PTSD, perceived job stress and resilience mediate the development of depression and alcohol use disorders following trauma exposure in firefighters. |  |
| Kim et al. (2015) [81] | CES-D, yet 21 point borderline version by Cho and Kim to distinguish between non-depression and depression | Continuous | b (SE) = 0.137 (0.219), Multiple regression analysis showed that fatigue is the most influential factor for depression, although this conclusion was based on a non-significant finding. Neither was the association between work stress and depression. | Fatigue | Call center employees who experienced great emotional stress appeared to have high levels of depression, and fatigue was a powerful factor influencing their depression. | convenience sampling used for recruitment, but no details given |
| Kitaoka‐Higashiguchi et al. (2002) [61] | SDS (Japanese version) | Continuous | ANOVA: Main effect of demands (F(1,683)=14.17, p<0.001) and the main effects of control (F(1.683)=41.75, p<0.001) were significant, but the interaction between both was not. Explanatory rates for demand and control were 4.8 and 9.1%. | Age | The tendency was observed that, irrespective of the level of control, the higher the demands of the job, the greater the SDS and vice versa. |  |
| Kolstad et al. (2010) [111] | Common Mental Disorder Questionnaire & ICD 10 | Categorized | High psychological demands (seldom as baseline) Sometimes OR = 1.13 (95%-CI: 0.71, 2.50); Often OR = 1.33 (95%-CI: 0.71, 2.50); Always OR = 4.17 (95%-CI: 1.93, 8.99) | Age, sex, previous depression, family history of depression, income, education, alcohol consumption, negative life events in previous 6 months and living alone | This study indicates that reporting bias inflates associations between high psychological demands and low decision latitude at work and the occurrence of depression, if studies rely on individual self-reports. |  |
| Koreki et al. (2015) [62] | CES-D | Continuous | Occupational stress and depressive symptoms: r = 0.52*** |  | The level of depressive symptoms was inversely correlated with the level of occupational satisfaction. In respondents who reported a moderate level of occupational stress, having fewer depressive symptoms was associated with higher occupational satisfaction, but this association was not significant in those who reported a high level of stress. |  |
| LaMontagne et al. (2008) [154] | doctor's diagnosis of depression (DSM-IV) | Categorized | Job strain population attributable risk (PAR) for depression was 13.2 % (95%-CI 1.1, 28.1) for males and 17.2% (95%-CI 1.5., 34.9) for females (effect size estimate for job strain in relation to depression (OR = 1.8] were extracted from published literature) | Occupational skill level | The numbers of compensated 'mental stress' claims compared to job strain-attributable depression cases suggest that there is substantial under-recognition and under-compensation of job strain-attributable depression. |  |
| Lee et al. (2012) [83] | CES-D | Continuous | Job demand ß: 0.173 (p=0.025); insufficient job control ß: 0.14 (p=0.049); interpersonal conflict ß: 0.188 (p=0.011), acculturative stress ß: 0,320 (p<0.001) | Gender, age, marital status, years of employment | Acculturative stress and work-related psychosocial factors significantly predicted 26.3% of the variance in depression. A path model revealed the mediating effect of job demand on the relationship between acculturative stress and depression |  |
| Lee et al. (2012) [82] | BDI | Continuous | Work stress and depression: r = 0.34*** |  | Work-related stress was positively associated with depression. Perceived stress was inversely related to self-esteem and positively associated with work-related stress and depression. | The results support the full mediation model by demonstrating that work-related stress indirectly influences depression through perceivedstress and self-esteem. |
| Li J et al. (2013) [101] | State-Trait Depression Scales | Continuous | Association between change of work stress between wave 1 and wave 2 and depressiveness at W3: log (effort/reward): Change score (increase per SD): 0.78 (95%-CI: 0.38; 1.18)***; overcommitment: 0.68 (95%-CI: 0.27; 1.09)*** | Age, gender, partnership, children, chronic diseases, medication, smoking, alcohol addiction, physical activity, work tenure, shiftwork, and weekly working hours | Negative changes in the psychosocial work environment, specifically increased ERI, are associated with depressive symptoms in German junior physicians |  |
| Li Q et al. (2019) [43] | CES-D (10-item Chinese version) | Continuous | Job stress/depressive symptoms: ß = 0.211 (95%-CI: 0.147; 0.276)** | Sex, age, education, SES, monthly income,  self-esteem | when migrant workers perceive a high level of social support, job stress is more likely to threaten their self-esteem and in turn increase their risk for depression |  |
| Lin HS et al. (2010)  [91] | BDI | Continuous | p < .001 job stress/depression scores | marital status, number of children, living with other people, health condition, monthly income | Depression scores were positively correlated with job stress and negatively with affective-oriented coping and social support |  |
| Lin TC et al. (2016)  [92] | Taiwan Depression Questionnaire | Continuous | Overall work stress /depression: ß: 0.80 (p < 0.001) | age, years of work experience, marital status, education level and having children | correlations of work stress with occupational burnout, as well as work stress and occupational burnout with depression level were all positive and significant |  |
| Liu et al. (2012)  [44] | CES-D (20-item Chinese version) | Continuous | Path coefficient for  Males: ERI-Ratio 0.439**; Overcommitment: 0.102*  Females: ERI-Ratio: 0.430**;  Overcommitment: 0.177** | Age, marital status, education | Both the effort/reward ratio (ERR) and overcommitment were significantly associated with depressive symptoms among male and female physicians. Psychological capital was a mediator in this association in women, only. |  |
| Looseley et al. (2019)  [120] | Harvard National Depression Screening Day Scale | Categorized | Association between work stress and depression not calculated |  | observed a high prevalence of perceived stress; 37% (95%CI 32–42%), burnout risk 25% (21–29%) and depression risk 18% (15–23%), and found that these issues frequently co-exist |  |
| Lunau et al. (2013)  [161] | CES-D (ELSA, HRS), EURO-D depression scale (SHARE) | Categorized | ERI/depressive symptoms: OR = 1.55 (95% CI: 1.27–1.89): low control/depressive symptom: OR = 1.46 (95%-CI: 1.19-1.79) | Sex, age, income, education, employment status, work time, heart disease, high blood pressure, stroke, diabetes, ≥1 Limitation in activities of daily living | Work stress is associated with elevated risk of prospective depressive symptoms among older employees in 13 economically  advanced countries (Europe, USA). Protective labor and social policies modify the strength of these associations. |  |
| Lunau et al. (2018)  [144] | CES-D | Categorized | RR_ERI_ = 2.11 (95%-CI 1.71–2.61),  RR_JobStrain_ = 1.84 (95%-CI 1.48–2.28) | Gender, age and education | Psychosocial work stressors increase, the risk of developing elevated depressive symptoms. There are no clear indication that internal or external resources buffer the association between psychosocial work  stressors and depressive symptoms. |  |
| Mackie et al. (2001)  [145] | CES-D | Continuous | Perceived work stress/depression: r=0.38 (p<0.01) (structural equitation analysis) |  | Increased exposure to employee involvement practices was indirectly associated with lower levels of depression through both perceived work stress and sense of coherence. |  |
| Kuhnke-Wagner et al. (2010)  [102] | ADS-K (German version of CES-D, short form) | Categorised | ERI/depressive symptoms_highest to lowest tercile_: OR = 4,44 (95%-CI 0.97-20.24)  Significant bivariate association between overcommitment and depression (p < 0.01) | Age,  sex, graduation, weekly working hours, decision latitude, work-privacy conflict | Higher risk for depressive symptoms among managers with an imbalance of high efforts and low rewards. The three reward dimensions were of different relevance (personal rewards showed the strongest association to depression, monetary the weakest). Less significant health restrictions by depressiveness are to be expected by reducing the imbalance of efforts and rewards at work. | *AK: mean of worktime: 45,1 h (SD ±10,8 h); ADS-K=general depression scale-short form; Informational bias: reporting bias (p.31, 3rd column, upper part) |
| Larisch  et al. (2003)  [103] | ADS (German version of CES-D) | Categorised | ERI-ratio/ depressive symptoms_highest to lowest tercile_:  OR = 5.94 (95%-CI: 2.45-14.36)  Overcommitment/ depressive symptoms:  OR = 5.92 (95%-CI: 2.92-11.99) | Age, gender, socioeconomic status, control at the work place, shift work | In this study, a strong correlation between the two components of the model of professional gratification crises, the ERI-quotient indicating situational aspects of the stress imbalance and the personal coping style of overcommitment as well as the existence of depressive symptoms in a group of middle-aged workers was proven. This connection found for the first time in Germany | * AJ: educational level and household income *AT: selection bias: women underrepresented; sample contains just one company; ADS = general depression scale; AF: first contact -> not reported |
| Mäntyniemi et al. (2012)  [116] | ICD-10 F32-34 (doctors diagnosis) | Continuous | Job strain/ disability pension due to depression: male: HR 1.24 (95%-CI: 1.00-1.53; female HR: 1.15 (95%-CI: 0.97-1.37) | Sex, age, job contract | High job strain is a risk factor for disability pension due to musculoskeletal diseases. No consistent association was found between job strain and the risk of disability retirement due to depression. | SES: Occupational position; bias: selection bias, reversed causality; AF: first contact -> not reported |
| McCleese et al. (2007)  [146] | CES-D | Continuous | Higher mean scores in distresses individuals (experience a vertical or/and horizontal plateau) (M=16.38, SD=10,50) than in the general population (M=9.25, SD=8.58); p<0.001 | - | Plateau-specific stress was higher than the stress experienced by the general population. Plateaued employees also reported more depression than the general population. Double plateaued employees reported higher depression than hierarchically plateaued employees. Content analysis revealed 27 distinct coping strategies reported by employees, representing 7 coping meta-themes. The most frequently occurring meta-themes were Discuss Problem, Job Withdrawal, and Mental Coping. Hierarchically plateaued employees reported slightly greater use of Mental Coping strategies than job content and double plateaued employees. | *AJ: educational level, marital status |
| Mezuk et al. (2011)  [147] | CES-D | Categorised | OR = 2.98 [95%-CI: 1.99–4.45] | Age, race, gender, marital status, education, labor force status, type of work, standardized net worth, smoking status, alcohol consumption | Job strain is associated with elevated depressive symptoms among older workers. In contrast to results from investigations of younger workers, job strain was unrelated to alcohol misuse | *AJ: educational level, marital status, race; AT: potential bias: Reporting + informational bias; AF: first contact -> not reported |
| Mino et al. (2006)  [63] | CES-D, GHQ-30 | Continuous | Not reported | **-** | A stress-management program based on the cognitive behavioral approach at the workplace may have potential for the prevention of depression. | AF: first contact -> not reported contact |
| Miyaki et al. (2012)  [64] | Kessler 6 (K6) scale | Categorised | Job strain/depression: OR = 1.096 [95%-CI 1.065-1.128]  Worksite Support to depression 0.837 (95%-CI 0.786-0891) | Age, sex, SES factors, years of education, management position, annual household income, energy-adjusted folate intake | Our cross-sectional study suggested an inverse, independent relation of energy-adjusted folate intake with depression score and prevalence of depressive symptoms in Japanese workers, together with the consideration of SES and job stress factors. | *AJ: years of education, income; AF: first contact -> not reported |
| Nakada et al. (2016)  [65] | SDS | Categorised | OR (95%-CI): Quantitative workload (high vs low): 3.81 (2.60-5.59); job control (low vs high): 2.85 (1.83-4.45); role conflict (high vs low) 9.27 (5.86-14.66); role ambiguity (high vs low) 6.10 (3.89-9.57); social support from supervisor (low vs high): 4.55 (2.96-7.01); social support from coworker (low vs high): 3.91 (2.56-5.97) | Age, sex, marital status, school category, overtime hours | We found that high role ambiguity, high role conflict, high quantitative workload, and low social support from family or friends were significantly related to depressive symptoms. To moderate role ambiguity and role conflict experienced by teachers, it is necessary to clarify the priority order of teachers’ work. | *AJ: educational level, marital status |
| Nitta et al. (2018)  [66] | SDS | Categorised | School principles: Quantitative workload OR = 6.62 (95%-CI: 2.63-16.70); role ambiguity OR = 4.94 (95%-CI: 1.57-15.53);  Vice-principles: social support from supervisors OR = 4.14 (95%-CI: 1.97-8.68), role ambiguity OR 9.71 (95%-CI: 4.08-23.14 | Gender, marital status, school category, overtime hours | Study confirmed the relationship between higher depressive scores and occupational stress in school principals and vice-principles. Quantitative work load & role ambiguity were predictive of depression in principles, role ambiguity & social support from supervisors in vice-principles | *AJ: educational level, marital status |
| Nourry et al. (2014) [119] | CES-D | Categorised | ERI: OR = 10.81 (95% CI: 5.1–23.0)*** | Gender, sports activities, smoking status, work seniority, work time, specialty area, work related happening over the previous 12 month (e.g., practical restructuring) | Among the nurse managers, a third had depressive symptoms, and 17,6% presented an effort-reward imbalance (ratio: ≥ 1). A significant association was found between depressive symptoms and effort-reward imbalance (OR = 10.81, 95% CI: 5.1–23.0, p < 10–3), and with esteem as a reward (OR = 3.21, 95% CI: 1.6–6.3, p < 10–2). | *AJ: Work seniority; AF: not mentioned if the Questionnaire was sent by post or via email |
| Park et al. (2008) [84] | CES-D | Categorised | Male: Job demands OR = 1.676 (95%-CI: 1.431-2.198), inadequate social support OR = 1.551 (95%-CI: 1.232-1.941); job insecurity OR = 2.015 (95%-CI: 1.607-2.392); lack of rewards OR = 1.878 (95%-CI: 1.482-2.367); occupational climate OR = 1.841 (1.491-2.279)  Female: Job insecurity OR = 1.946 (95%-CI: 1.419-2.698); organizational injustice OR = 1.621 (95%-CI: 1.140-2.299); Occupational climate: OR = 1.782 (95%-CI: 1.295-2.487) | Male: age, marital status, employment type, job stress  Female: age, marital status, education level, employment type, change in work content, job stress | These results indicate that job stress may play a significant role in increasing the risk of depressive symptoms, and that further preventive efforts and research are needed to reduce job stress and address health problems caused by job stress among Korean employees. | *AT: recall and response bias are possible; AF: first contact -> not reported |
| Peter et al. (2016) [104] | BDI-V | Continuous | ERI to zu depressive symptoms t1  Women ß: 0.091 (p < 0.01),  men 0.083 (p < 0.01) | Age, neg. affectivity, OC, depressive symptoms at baseline, SSI, occupational position, full- & part-time work | ERI and WFC increase the risk of future episodes with depressive symptoms in men and in women irrespective of SSI, occupational position, full- or part-time work, regional factors or individual characteristics. | *AJ: Occupational position, education and training, status inconsistency; AF: first contact -> not reported |
| Proeschold-Bell et al. (2013) [148] | PHQ–9 | Categorised | OR = 1.81 (95%-CI: 1.23; 2.66)** | Life unpredictability  social isolation, guilty about work, doubting call, ministry satisfaction, financial stress, status control-next appointment, age, gender, race, job position, hours worked, age, time in ministry, female, black, another race, district superintendent, deacon. Local pastor, hours worked | High prevalence rate of depressiveness among clergy (11.1 %) were significantly associated with job stress and other extrinsic and intrinsic demand and reward variables consistent to effort-reward imbalance theory |  |
| Pulkki  -Raback (2015) [117] | BDI-II | Continuous | Base model: Demands ß: 1.01***  Control ß: -0.07 (n.s.)  Model with emotional adversity in childhood as moderator:  Demands ß: 2.49***  Control ß: 0.06 (n.s.) | Age, gender, depressive symptoms in former wave, adversity, different interaction terms | High job demands are more strongly related to depressive symptoms among employees with severe emotional adversities in childhood. |  |
| Raskin et al. (2014) [129] | CES-D | Continuous | Model 2: Perceived job resources b (SE) -0.14 (0.07)*; Perceived job control 0.00 (0.07) (n.s.) | Different forms of coping, percent of vacation days taken, day of vacation given, wage, number of children in the group, different proportions of children in the group, hours worked last 8 days, tenure in this child care facility | Very high prevalence rate of depressiveness found among caregivers (54 %). The finding that higher perceived job resources (e.g., satisfaction with performance and work  rewards) were associated with fewer depressive symptoms is particularly encouraging, given that  job resources can be enhanced by interventions |  |
| Rayens et al. (2013) [149] | CES-D | Continuous | Perceived job stress:  Men: ß: 0.89***  Women: ß: 1.23***  Satisfaction with farm work:  Men: n.s.  Women:  ß: -1.13** | Number of farm work hours in last week (actor & partner);  satisfaction with farm work (actor & partner);  perceived stress (partner); number of health conditions (actor & partner); minority, age, income | Work related stress and depression, although separate constructs, are intrinsicately linked among aging farm workers in household. |  |
| Rösler et al. (2008) [105] | ADS-K | Continuous | Standardized path coefficient:  Decision latitude (n.s.)  Demands: 0.21**  Job security: -0.42***  status related rewards :0.23*  social rewards: -0.33** | - | Employees with high job demands/effort, low job security, low social recognition reported higher depression scores. Unexpectedly, status-related rewards were positively associated with depression, while no significant effects for decision latitude was found. |  |
| Rugulies et al. (2006) [112] | Five-item Mental Health Inventory (MHI-5) of the 36-item Short-Form Health Survey | Categorised | Women  low influence at work: RR = 1.96 (95%-CI:  1.10; 3.47); low supervisor support: RR = 1.92 (95%-CI: 1.13; 3.26)  Men  job insecurity:  RR = 2.09 (95%-CI: 1.04- 4.20) | Age, family status, school education, change in employment status,  depression score at baseline, smoking, alcohol consumption, leisure-time physical activity, socioeconomic position | Psychosocial work characteristics contribute to the development of severe depressive symptoms in the Danish workforce. Different aspects of the psychosocial work environment seem to be important for men and women. |  |
| Saijo et al. (2014) [67] | PHQ-9 (Japanese version) | Categorised | Job strain_high vs low:_ OR = 3.43 (95%-CI: 1.72-6.85); support from co-workers: OR = 0.91 (95%-CI: 0.82-0.99); support from supervisor: (n.s.) | Gender, age, marital status, clinical experience, hospital vs. clinic, location (large city to town), specialty, days off per month, night duty shifts per month, on call shifts per month, working hours per week | High job strain was related to depressive symptoms, support from co-workers had a buffering effect on depressive symptoms |  |
| Saijo et al. (2014) [68] | PHQ-9 | Categorised | Men:  job demands: OR = 1.92 (95 %-CI 1.34; 2.74), job control: OR = 2.30 (95 %-CI 1.52; 3.47),  social support at work: (OR = 1.82 (95 %-CI:1.16; 2.86)  Women:  job demands: OR = 1.64 (95 %-CI: 1.05; 2.58),  job control: OR = 2.24 (95 %-CI 1.33; 3.75) | Age, marital status, employment grade, shift work, hypnotic use, social support from family/friends | There were more-than-additive interactions  of job control and social support at work on depression in men |  |
| Saijo et al. (2007) [69] | CES-D | Categorised | Variance in  workload_high vs. low_: OR = 2.05 (95%-CI 1.29; 3.25),  intergroup conflict_high vs. low_: OR = 1.91 (95%-CI: 1.26; 2.88), role conflict_high vs. low:_ OR = 1.87 (95% CI: 1.24; 2.80), self-esteem: OR _low vs. high:_ 5.78 (95%-CI 3.93;8.50),  job control: OR _middle vs. hig_: 0.64 (95%-CI: 0.43; 0.94) | Gender, age, marital status, smoking, drinking, job class, type of job, quantitative workload, cognitive demand, intra group conflict, role ambiguity, social support from supervisor or coworker or family/friends, non-work activity,  self-esteem | High variance in workload, high intergroup conflict, high role conflict, and low self-esteem were significantly related to depressive symptoms. | Small proportion of female subjects in the study sample |
| Saijo et al. (2016) [70] | CES-D | Categorised | Job demand OR = 1.39 (95%-CI: 1.17; 1.66)***; Job control OR = 0.63 (95%-CI: 0.3; 0.74)***  Job strain (high demand and low control vs. low demand and high control) OR = 4.31 (95%-CI 2.83; 6.59); Support from supervisors: OR = 0.81 (95%-CI: 0.68; 0.98)*  Support from co-workers: OR = 0.89 (95%-CI: 0.74; 1.06) | Age, gender, education, marital status, income, job type, job rank, working hours, night shift, support from family and friends | Significant relationships between job demands, job control and support from supervisors on depressive symptoms were found. Job control was most related to depressive symptoms. |  |
| Sakata et al. (2008) [71] | CES-D (Japanese version) | Categorised | ERI-Ratio OR = 8.83 (95%-CI: 2.87; 27.12); Overcommitment OR = 1.99 (95%-CI: 0.95; 4.14) (n.s.); low social support score: OR = 2.77 (95%-CI: 1.36; 5.64) | Age, gender, year of residency, frequency of overnight work, alcohol consumption, exercise, smoking, overcommitment, social support | This study suggests that balancing effort and reward and having adequate social support at workplaces may be important factors for improving depression. |  |
| Sandström et al. (2012) [123] | Montgomery – Asb scaleerg depression rating scale+ Hamilton scale | Continuous | The correlation overview for the only signiﬁcant behavioral PLS LV1 (p < 0.005).  The correlation between brain scores and reaction times for the 2-back task within LV1. The correlation was signiﬁcant (r = 20.73) | - | The present findings suggest a difference between patient categories, such that long-term stress relative to acute depression induces changes in functional brain activity, notably in areas within the frontal cortex, as well as a flattening of the diurnal cortisol curve. |  |
| Santa Maria et al. (2017) [106] | PHQ-4 | Continuous | Job demand to emotional exhaustion: ß: 0.93**; emotional exhaustion to depression & anxiety: ß = 0.55**  Job resources to depression and anxiety: ß = -0.30** |  | Job demands predicted emotional exhaustion, which in turn predicted depression. Job resources, operationalized as social  support by colleagues, shared values, and a positive leadership climate,  were negatively associated with self-reported depression and anxiety levels and  buffered the impact of job demands on emotional exhaustion | Not possible to disentangle the effect of demand and resources on depression by the given information (combined endpoint of depression and anxiety in the analysis) |
| Shang et al. (2015) [45] | PHQ-2 | Categorised | OR = 3.55 (95%-CI: 2,49; 5,07) | Age, Gender,  Marital status,  subjective socioeconomic status, smoking  alcohol drinking  physical exercise | Results confirmed the well-established association between the  work stress model in terms of ERI and depressive symptoms | A significant modifying effect of SOC was found in addition |
| Shen et al. (2014) [46] | CES-D | Continuous | ERI-Ratio: ß = 0.372**  OC: ß = 0.148**  After adjusting for psychological capital:  ERI-Ratio: ß = 0.242**  OC: ß = 0.135** | Gender, age, marital status, education  (Psychological capital: mediator) | Both extrinsic stress (ERR) and  intrinsic stress (over-commitment) was positively associated  with depressive symptoms, psychological capacity partially mediated the effect of occupational stress on depressive symptoms |  |
| Shepard-Binigan et al. (2016) [150] | CES-D | Continuous | Job stress: ß = 1.73 (SE: 0.37)**;  Work from home: ß =  -1.36 (SE: 0.51)**;  3-4 days work from home ß = -5.11 (SE: 2.44)** | Hours worked per week, work schedule, schedule flexibility, work from home, number of hours work from home | Results show that  job stress may worsen depressive symptoms and that working  from home may improve depressive symptoms among working  women with young children. |  |
| Siegrist et al. (2012) [162] | CES-D (short & long version), EURO-D | Categorised | Cross-sectional/longitudinal OR (95%-CI): Depressive symptoms vs ERI: USA 2.28 (1.59; 3.28)***/n.s.; Europe 1.97 (1.75; 2.23)***/1.51 (1.28; 1.78)***; Japan 1.64 (1.02; 2.63)*/N/A Depressive symptoms vs low control: USA 2.26 (1.57; 3.25)***/n.s.; Europe 1.66 (1.45; 1.90)***/1.42 (1.20;1.68)***, Japan n.s./N/A | Gender, age, education, employment status, working hours (Longitudinal: + depressive symptoms wave 1) | work stress is significantly associated with depressive symptoms in cross-sectional and, in part, in longitudinal analyses across different regions of the world |  |
| Simmons et al. (2009) [151] | indicator including two domains of depression in the last  month: feeling ‘‘down, depressed, or hopeless’’ and ‘‘little interest  or pleasure in doing things’’. | Categorised | OR (95%-CI):  Whole sample: physical demands: 1.25 (1.12–1.40); psychological demands: 1.59 (1.33–1.89); decision latitude: 0.74 (0.64–0.86); flexibility: 0.19 (0.11–0.32); supervisor support for work and family: 0.73 (0.66–0.81); coworker support: 0.58 (0.48–0.69); job insecurity: 3.01 (2.02–4.50) (all p < 0.001); yet, there were differences in the association between working poor (job security*) and working non-poor (support from supervisor** and coworkers**) | Bivariate analysis, only | All psychosocial job characteristics demonstrated significance at p < 0.001 in the overall sample. Differences in certain associations between working poor and non-poor were found |  |
| Smith et al. (2012) [152] | Composite International Diagnostic  Interview-Short Form for Major Depression (CIDI-SFMD) | Categorised | Increase in psychological demands: OR = 2.36 (95%-CI: 1.14, 4.88)*;  change in job control n.s.; change in job strain n.s.; change in social support n.s. | Gender, age  group, marital status and presence of children < 12 y,  occupation change, level of education, baseline levels  of job strain and social support, presence of chronic  health conditions, having subclinical depression in  2000 or 2002, family history of depression,  personal history of depression. | Focusing on the components of job strain, we found an elevated risk of depression only for negative changes (increases) in psychological demands. |  |
| Stansfeld et al. (2012) [121] | University of Michigan  version of the CIDI | Categorised | Job strain_high vs low_ Phase 1 OR 1.72; 95% CI=1.16, 2.57, p=.008; Phase 2 OR 1.76; 95% CI= 1.16, 2.67, p=0.007; Phase 3 OR1.96; 95% CI=1.28, 3.00, p=.002 | Age, gender | Repeated exposure to job strain is associated with increased risk of major depressive disorder. |  |
| Steinhardt et al. (2011] [163] | CES-D | Continuous | Mediation: Work stress >> burnout >> CES-D  Total effect b = 0.44, p < 0.001; direct effect: b = 0.13, p < 0.05;  Indirect effect b=  = 0.32 p < 0.001  R² = 0.43 | Years taught, teaching award, high school, gender, minority | Chronic work stress was strongly associated with both emotional exhaustion and depressive symptoms. The three cardinal symptoms of burn out mediated the association between work stress and depressive symptoms.  The total effect of stress on depressive symptoms, taking together the direct and indirect effects via burnout, accounted for 43% of the total  variance. | For indirect paths, this analysis produces  point estimates and three varieties (percentile, bias corrected,  and bias‐corrected and accelerated) of  bootstrapped 95% confidence intervals.  reversed causality, reporting bias and selection bias could have limited the results. |
| Takaki et al. (2010) [72] | CES-D (Japanese version) | Continuous | Effect (95%-CI) of job strain index on depression:  Men: Total effect: 0.218 (0.146, 0.291); Direct effect: 0.112 80.042, 0.182); Mediation effect: 0.1.06 (0.075, 0.142)  Women: Total effect: 0.180 (0.118; 0.242); Direct effect: 0.113 (0.055, 0.171); Mediation effect: 0.067 (0.043, 0.094) | Age, working hours per week, career, position, annual household income, current smoking, alcohol consumption, workplace social support | Total effect of job strain index on depression or sleep disturbance were all positive and significant (p<0.05) in both genders. All the effects of job strain index on workplace bullying and all the effects of workplace bullying on depression or sleep disturbance were positive and significant (p<0.05) in both genders |  |
| Tatsuse et al. (2019) [73] | CES-D | Categorised | OR (95%-CI) work controll_ow vs high_ 1.66 (1.13–2.44); job demand_high vs low :_ 1.79 (1.24–2.63); support at work_low vs high_ 1.11 (0.76-1.61) | Age, sex, marital status, depression at baseline, job satisfaction, working long hours. shiftwork, sleep duration, chronic illness. | Job  satisfaction, and psychosocial stress at baseline predicted development of  and recovery from depression at 1-year follow up, respectively. |  |
| Theorell et al. (2014) [124] | Depressive mood measured with the Symptom Check List-Core Depression (SCL-CD) | Continuous | Job strain:  Men  B = 1.138 (95%-CI: 0.670; 1.606)  Women  b=0.847 (0.410 to 1.284)  p<0.001 | age, income, depressive symptoms at baseline | In Sweden, job strain was as strongly  related to depressive symptoms among men as among women. |  |
| Tomioka et al. (2011) [74] | CES-D | Categorised | OR (95%-CI) ERI-Ratio_upper vs lower tertile_ in the  short working hours group  0.6 (0.2–1.8),  middle working hours group  8.5 (3.0–24.0) ,  long working hours group 9.9 (3.8–25.7);  OR (95%-CI) OC_upper vs lower tertile_ in the  short working hours group  10.4 (3.3–32.5),  middle working hours group  4.3 (1.6–11.8),  long working hours group 2.6 (1.1-6.3) | gender, years of clinical experience, social support | The association between occupational stress due to effort–reward imbalance  and overcommitment showed variation depending on the duration of working hours |  |
| Tsuboi et al. (2006) [75] | CES-D | Continuous | Job stress participants exhibited significantly higher CES-D scores than low stress participants (t =3.34, p < 0.005) | No | Participants with high JS exhibited higher depressive symptoms and a lower level of plasma LDL+VLDL |  |
| Tsutsumi et al. (2001) [76] | CES-D | Categorised | low control: OR = 4.71 (95%-CI: 1.61-13.72), effort reward imbalance OR = 4.13 (95%-CI: 1.39-12.28), OC: OR = 2.56 (95%-CI: 1.01-6.47) | Age, gender, occupational status, job type, job characteristics | This study confirms that the two job stress models identify different aspects of stressful job conditions. Moreover, effort-reward imbalance and low control at work are both associated with symptoms of depression |  |
| Tsutsumi et al. (2011) [77] | CES-D | Categorised | OR (95%_CI): ERI: 3.57 (2.43; 5.26); organizational rewards: 5.14 (3.36–7.92); esteem-related rewards: 2.81; 2.00–3.95; monetary rewards: 2.40 (1.70–3.39) | Age, gender, number of physicians, number of beds, number of stuffs, number of patients per day, working hours, sleep hours | component most strongly associated with depressive symptomatology was ERI with organizational reward, following by ERI with esteem-related reward and ERI with monetary reward |  |
| Vearing and Mak (2007) [155] | CES-D (11-item abridged adaption) | Continuous | b (SE): log ERI: 0.72 (0.26); OC: 0.29 (0.25); the addition of OC and log of ERI ratio (along with N and C from the first step) explained a significant additional 11.4% of the variance in depressive symptoms (out of a total of 44.2% explained), F (3, 201) = 13.66 (p < 0.001) | Gender, age, Big Five Inventory | Regression analysis of depressive symptoms revealed a medium effect of neuroticism (N), followed by small effects  of workplace social support, conscientiousness (C), and ERI ratio, accounting for 44% of the variance in  depressive symptoms and providing support to the utility of considering both big five and work stress factor |  |
| Wallace (2006) [133] | CES-D (modified form) | Continuous | b (SE): JOB DEMANDS: Overload: 1.007 (1.468)***; hours at office: n.s.; hours at home: n.s.; extra activities: 0.193 (0.289)**; JOB CONTROL over: Flexibility: -0.73 (-0.147)***; number of hours: -0.22 (-0.050)*; SOCIAL SUPPORT: Coworker: 0.307(0.399)*; organizational: n.s.; SIGNIFICANT INTERACTIONS: Overload X coworkers: -0.111 (-0.686)***; hours at office X coworkers: 0.004 (0.344)*; Extra activities X coworkers: -0.068 (-0.353)**; | Adequate income, law experience, sex, housework hours, no. of children, preschool children, partner works; social and emotional support by spouse, different interaction terms | Work overload (component of 'demand') was positively, flexibility (component of control) as well as organizational support were negatively associated with depression, also significant interaction between different components |  |
| Wang C et al. (2016) [47] | PHQ-9 | Continuous | Direkt Effect: ß (95%-CI): Job burden: 0.19 (0.09; 0.29)***; Capital: -0.34 (-0.42; -0.26)***; Indirect effect: ß (95%-CI): Job burden: 0.06 (0.02; 0.10)**; Capital: -0.02 (-0.03; -0.01)** | (Influence of gender, age, education level, marital status and job position separately tested for model fit) | Job burden and capital are directly associated with depression (and wellbeing). Personality can mediate the relationship among job burden, capital, depression (and well-being). For occupational groups the theoretical model has general applicability among populations |  |
| Wang J et al. (2010) [134] | CIDI-SFMD | Categorised | OR (95%-CI) (combined models): Job strain ratio (>1 vs <=1): 1.46 (1.15; 1.85); negative life events :1.90 (1.51; 2.40); chronic stress: 1.60 (1.13; 2.27); childhood traumatic events: 1.43 (1.12; 1.83); | gender, age, marital status, education and time-varying  covariates (employment status, self-rated health and having  one or more long-term medical conditions) | Any one type of psychosocial factors may not be a sufficient cause for MDE. The combination of various stressors may be one of the causes of MDE. The risk of developing MDE is increasing with the number of stressors. No interaction between stressors found. |  |
| Wang J et al. (2009) [135] | CIDI-SFMD | Categorised | OR (95%-CI): Low job strain, no change over time: 1.00; High job strain, no change: 1.52 (1.00; 2.30); High to low job strain: 0.97 (0.61; 1.53); Low to high job strain: 1.60 (1.00; 2.57) | Adjusted for gender, age, educational level, status of major depression  from 1994–1995 to 2000–2001, perceived health status, and  childhood traumatic events at baseline | Similar low risk of depression for those who had a change from high to low job strain with those having constantly low stress. Those exposed to persistent high job strain, only those who reported good or excellent health at baseline had a risk of major depression |  |
| Wang J et al. (2011) [136] | PHQ-9 | Categorised | OR (95%-CI) job strain: 2.25 (1.51; 3.34); ERI: 2.26 (1.47; 3.46); WFC: 1.45 (1.29; 1.63) | Age, marital status, educational level, personal income | Job strain, ERI, Work-family conflict are equally important in their relations to depression. Moreover, there may be effect modifications among stressors inside & outside of the workplace in the relation to the risk of depression | Forms of bias: Selection, recall and reporting bias |
| Wang LJ et al. (2011) [93] | Taiwanese Depression Questionnaire (TDQ) | Categorised | OR (high vs low; 95%-CI): Job control: 0.11 (0.04-0.31)***; Work demands: 2.46 (1.02; 5.93)*; Social support: 0.11 (0.02-0.55)** | Age, gender, position, geography, marital status, department, working hours, night shift duty days, smoke, alcohol, exercise | Job stress (Karaseks job strain model) plays an important role in depression in physicians | Bias: Selection and information bias |
| Wang SM et al. (2015) [94] | TDQ | Continuous | ß: 0.68 (p<0.001); R² = 0.51 | Marital status, work unit, working shift | Higher stress levels and lower personal resourcefulness in psychiatric nurses were correlated with higher depression levels. Work stress was found to be a significant predictor for depression in these psychiatric nurses. | Selection bias |
| Wege et al. (2018)  [107] | Doctor’s diagnosis | Categorised | RR (risk ratio) (95%-CI): ERI (men): 1.82 (1.36; 2.44)***; ERI(women): 1.88 (1,51; 2,33)***; Overcommitment (men): 1.16 (1.10; 1.23)***; Overcommitment (women): 1.09 (1.04; 1.12)***; Effort (men): 1.08 (1.01; 1.16)**; Effort (women): 1.07 (1.01; 1.13)*; Reward (men: 0.94 (0.91; 0.97)***; Reward (women: 0.93 (0.91; 0.96)*** | age, marital status, education, income, employment, smoking, alcohol  consumption, physical activity, and BMI at baseline, chronic diseases at baseline | This study reports associations of similar consistency and strength of a stressful psychosocial work environment, as defined by the effort-reward imbalance model, with risk of reported depression between men and women | Selection bias |
| Wu et al. (2011) [48] | CES-D (Chinese version) | Continuous | Relationship to depressive symptoms (structural equation coefficient): Role overload: 0.200**; Role insufficiency: 0.186**; Role ambiguity: n.s.; Role boundary: 0.125**; Responsibility: n.s.; Social support: -0.172**; Rational coping: -0.105** | Age | Role overload, role insuffiency and role boundary were significantly associated with depressive symptoms in female nurses. These effects were mediated by social support & rational coping | Informational bias |
| Yoon SL, Kim JH (2013) [85] | CES-D (Korean version) | Categorised | OR (95%CI): High job demand: 1.28 (0.80; 2.05); insufficient job control: 0.88 (0.50; 1.57); inadequate social support: 0.99 (0.57; 1.70); job insecurity: 1.60 (1.10; 2.32)*; organisational injustice: 0.66 (0.35; 1.26); lack of reward: 1.99 (1.07; 3.70); discomfort in occupational climate: 1.52 (0.87; 2.66); surface acting: 2.46 (1.56; 3.86); deep acting: 1.18 (0.73; 1.92) | Age, marital  status, position, type and years of employment, and monthly salary | The results of this study provide evidence that nurses are at high risk for having job-related stress and depressive symptoms | Bias: response, selection |
| Yoshizawa K et al. (2016) [78] | CES-D | Categorised | OR (95%-CI): Role conflict: 1.40 (0.52; 3.74).; Role ambiguity: 2.45 (0.85; 7.05); Job control: 0.36 (0.13; 0.97); Social support supervisor: 0.18 (0.05; 0.65); Social support coworkers: 1.20 (0.41; 3.53); quantitative workload: 5.18 (1.34; 19.97); Variance in workload: 2.01 (0.48; 8.30); occupational hazards: 1.79 (0.65; 4.97) | Gender, marital status, children, health practice index, length of work in psychiatric department (years) | our study revealed that age, the HPI score, job control, social support from a supervisor, and quantitative workload were associated with depression in Japanese psychiatric nurses. | Bias: selection bias |
| Li W et al. (2019) [49] | Self-created 5 item scale on how often feeling sad, losing self-confidence, encountering insurmountable difficulties, being not able to get going or daily life being affected due to emotional problems in the last four weeks | Continuous | Work stress: b (SE): 1.115 (0.107)**;  Stress X community supportive network b(SE):-0.281 (0.101)** | Age, gender, education, self-rated health, self-rated social class, community supportive network (moderator1), community cohesion (moderator 2), community composition (moderator 3) | Rural–urban migrant workers in China experienced high work stress and high depressive symptoms. Community supportive networks moderated the relation between work stress and  depressive symptoms. | Limitations due to secondary data analysis; community variables were assessed through individuals’ perception; reverse causality |
| Kim et al. (2020) [137] | CES-D (16-item scale without positive affect items) | Continuous | emotional demands of MBO vs professionals (β, 0.159, n.s.); buffering effect (interaction with emotional demands) of job autonomy: β = 0.021 (p<0.05), job satisfaction: β = -0.027; (p<0.01), and job security: β = -0.070; (p<0.001) | Age, sex, marital status, years of immigration, previous education, Canadian education, annual income, working hours, language barriers | The benefits of job satisfaction and security may protect MBOs from the adverse mental health effects of job stress. | Study population First generation Korean immigrant microbusiness owners,  professionals, office workers, and manual workers |
| Sun et al. (2020) [50] | 5-item scale developed by the Chinese CLDS research team | Continuous | ß(SE) = 0.32 (0.01)** | Age, sex, having a spouse, education, self-rated class, self-rated health, medical benefit, rural, urban, migrant | Stressors from work, family and community comprised a general model that explains depressive symptoms in Chinese older workers | Not clear how participants were approached. Self-rated class was also collected. Older workers, so healthy worker survivor bias may be in play. |
| Åhlin et al. (2018)  [125] | SCL-CD, short version with 6 items of the brief subscale from the Hopkins Symptom  Checklist (SCL-90) | Categorised | High job strain: OR = 1.35; (95%-CI: 0.78; 2.37)  -analysis based on trajectory modelling for those with previous depressive symptoms | Previous depressive symptoms, No of children at home, civil status (married, cohabiting) | The results indicate that the levels of job  demands and control were relatively unchanged across  6 years and suggest that long-term exposure to a high strain  or active job may be associated with increased risk for subsequent  depression. |  |
| Magnusson Hanson et al. (2014)  [126] | SCL-CD_6_., short version with 6 items of the SCL-90 | Continuous | Total effect: Work demands: b=0.114 (95%-CI: 0.068; 0.160); workplace support: b =-0.103 (95%-CI: -0.125;-0.081)  indirect effect: demands b = 0.013 (95%-CI: 0.005;0.020), workplace support: b =-0.006 (95%-CI: -0.014; 0.002) |  | The higher demands at work might cause an increase in depressive symptoms, in part, by increasing disturbed sleep, although the mediated effect was relatively small compared to the total effect | **Possible Bias: informational bias** |
| Magnusson Hanson et al. (2009) [127] | brief subscale from the Hopkins Symptom Checklist (SCL-  90, Lipmann 1986) | Continuous | Demands: b = -0.05, SE: 0.01 (p<0.001 in men; n.s. in women); decision authority:(b = -0.09, SE 0.04 (p<0.05) in men; b = -0.15, SE 0.04 (p<0.001) in women); support from superiors: n.s. in men; b = 0.08, SE 0.03 (p < 0.05) in women; support from fellow workers: n.s. in men; b = -0.11, SE 0.03 (p<0.01) in women | Depressive symptoms at baseline, age, marital status, birth country, labor market sector, income at baseline and  employment status at follow-up | study supports the theory that decision  authority, support and conflicts at work are predictive of  depressive symptoms in the general Swedish working  population | possible biases: proxy measures (misclassification); no baseline exclusion of depressed employees; SCL-6 depression subscale used was not validated in this population; both working conditions and  depressive symptoms were measured by self-completion  questionnaires which can lead to “common method variance”; no adjustment for personality factors |
| Schramm et al (2020) [108] | HRSD-24 (doctor’s diagnosis); BDI-II | Continuous | Significant improvement in mean rewards (W-IPT: TAU = 27.1 vs. 21.7; p < 0.05) and significant reduction on ERI-Ratio (W-IPT: TAU = 1.1 vs. 1.7; p < 0.05) | - | A work-focused IPT program for the treatment of depression associated to work stress was feasible and highly acceptable. W-IPT turned out to be more effective than standard treatment in reducing depression and work-related problems (ERI !) |  |
| Wu et al. (2021) [51] | CES-D (Chinese version) | Continuous | Before fishing (ρ = 0.48, p < 0.01) and after fishing (ρ = 0.57, p < 0.01); interaction work stress × low hair cortisol was a significant predictor of depression symptoms b = 1.45 (p < 0.001), but interaction work stress × high hair cortisol n.s..; significant moderating effect of hair cortisone (regression coefficient β = 0.65 (p < 0.001) |  | Fishermen with low hair cortisol or high hair cortisone were more likely to show depressive symptoms after experiencing stress, and were less likely to show depressive symptoms when faced with lower levels of work stress, consistent with the differential susceptibility model. | Possible bias: recall bias, selection bias |
| Åhlin et al. (2021)  [131] | Symptom Checklist-core depression (SCL-CD6) scale (SLOSH-study)  MDI (WEHD-study) | Categorised (regarding the association between work stress and depression) | Total effect: RR=1.88, (95%-CI:1.45; 2.31) (SLOSH), RR = 1.64, (1.18; 2.11) (WEHD), | age, sex and panel (SLOSH data), education, cohabitation, physically strenuous work and chronic diseases | This study supported a prospective association between job demands and (incident) major depression, that appeared mainly direct or through other pathways than via LBP. In SLOSH, the findings indicted that part of the association was attributable to interaction and/or mediation, while these results were not supported in WEHD | Possible bias: self-report, misclassification of the mediator |
| Jung et al. (2020) [86] | CES-D (Korean version) | Continuous | Job stress had no direct significant effect on depression (ß= 1.556, n.s). Social support had a significant mediating effect between work stress and depression. Job stress was significantly inversely associated with social support. ß = –3.407 (p < 0.01), which was significantly inversely associated with depression ß = –3.314 (p<0.001). | Emotional labor, resilience | The emotional labor of nurses with long working hours influenced depression, whereas job stress did not.  Resilience had a negative mediating effect on the relationship between emotional labor and depressive symptoms. Social  support had negative mediating effects on the relationship between job stress and depression. | Limited representativeness to smaller hospital (≤ 300 beds); some potential confounders not included in analysis (e.g., SES, past medical history and family history) |
| Beschoner et al. (2021) [109] | BDI (BDI-n in 2006, BDI-II in 2016) | Categorised | - | - | Data show changes in workplace stress and mental health in psychiatrists in a decade in which a reduction in working hours has been required by law. Working hours and free weekends were associated with mental health indices. Correlation analyses showed that a reduction in weekly working hours and working days at weekends was related to reduced scores for effort-reward-imbalance, burnout and depression. Due to the different versions of the BDI, no comparisons can be calculated between 2006 and 2016 for depression prevalence. | No results for depression due to different measures at both times |
| Yang et al. (2020) [52] | CESD-10 | Categorised | b=0.16, ß= 0.17 (p < 0.001) (direct path); b = 0.20, ß =0.21 (p < 0.00)1, PM=55.56% (95 %-CI: 0.16; 0.26) (for indirect effect, via stress and fatigue) | Age, marital/cohabitation status; nature of work (mental and/or physical oriented) | The direct path from workaholism to depressive symptoms was significant and positive; workaholism was indirectly associated with depressive symptoms through  increasing stress and fatigue; fatigue had a greater mediation effect than stress |  |
| Weigl et al. (2021) [110] | BDI-II | Continuous | Work dissatisfaction: ß=0.237 (95%-CI: (0.194; 0.280); Work overload: ß =0.161 (95%-CI: 0.124; 0.198) | No of sick days during the last 12 month, average working time, overtime hours, occupational group, educational level, previously diagnosed psychiatric disorder, examination status | Different aspects of chronic work stress seem to be associated with levels of depression in nurses. Most important work-related predictors of depression scores in nurses were ‘Work dissatisfaction’ and ‘Work overload’ | few details on methods |
| Mohamed AF et al. (2022) [88] | Depression Anxiety Stress Scales (DASS) | Continuous | Mean Score Difference (95%- CI) before and after intervention:  Stress: -6.00 (-7.77; - 4.23)***  Depression: -4.14 (-5.88; -2.39)*** | - | The findings of this study reported that the WHP  intervention program appeared to be beneficial in improving  self-perceived stress, anxiety and depression, health-related  quality of life and coping skills of the employees. | Hawthorne effect through self-perceived working stress |
| Mayerl et al. (2020) [132] | EURO-D depression scale | Continuous | See publication | - | Employees who perceive higher levels of ERI in the workplace are more likely to also experience higher levels of depressive symptoms; within-person increases in ERI go along with within-person increases in depressive symptoms at the same point in time. Yet, no lagged effects of ERI and depressive symptoms were found at the within-person level. Such an effect was only found at the between person level. |  |
| Yong et al. (2020) [53] | Self-Rating Depression Scale by Sirodff (1977) | Continuous | In the bivariate model ERI was significantly associated with depressiveness (p<0,001). In the multiple model ERI was not significantly associated with depressiveness after adjusting for burn-out (yet, ERI was significantly associated with burn out, which was significantly associated with depressiveness) | Sex, age, working years, level of education, work type, marital status, working shift, annual income, Job burn-out score | A higher degree of occupational stress was associated with poorer mental status |  |
| Hsieh et al. (2021) [95] | CES-D | Continuous | In the bivariate analysis work stress was significantly correlated to depressiveness (r = 0.456, p < 0.001); in the structural equation model total effect from stress to depressive symptoms significant (z = 3.125, p < 0.001), but direct effect n.s.; mediating effect of sleep (z = 2.931, p < 0.001) and burnout (z = 3.285, p < 0.001), | - | Work stress did not affect depressive symptoms directly, but it affected depressive symptoms through the mediating effects of quality of sleep and occupational burnout. |  |
| Mohamed MY et al. (2023) [158] | SCID I for diagnosis of depression according to the DSM-IV; BDI | Categorised | - | - | Medical residents have remarkable work stress that has its impact on their psychological health in the form of anxiety and depressive symptoms that may affect their quality of life and the quality of services they provide to their patients. | Stress within the medical residents should be thoroughly identified in order to cease them or decrease their negative impact on the health and their outcome. |
| Kploanyi et al. (2020) [159] | CES-D | Categorised | Association between work stress and depression n.s. | Age, sex, marital status, dependents, employment, job ranking, hours per week, work experience, alcohol intake, | This study did not find a statistically significant relationship between depression and occupational stress | Reporting bias,  Reverse causality |
| Almroth et al. (2022) [128] | Diagnosis taken from in-and out-patient registers using ICD definition (F32 & F33) | Categorised | Men: Job control _low vs high_: HR (95%-CI)=1.43 (1.39;1.48); Job demands_high vs low_: HR (95%_CI)=1.23 (1.20; 1.26); Job strain _high vs_:  _low_: HR (95%-CI)= 1.26 (1.23; 1.30)  Women: Job control _low vs high_: HR (95%-CI)=1.27 (1.24; 1.30); Job demands_high vs low_: HR (95%-CI) = 0.83 (0.8; 0.86); Job strain _high vs low_ HR (95%-CI)= 0.99 (0.96; 1.01) | Birth year, birth country, civil status, number of children, previous psychiatric diagnosis, parental occupation, parents’ psychiatric diagnoses, education, mutually adjusted for job demands and control | Lower job control, measured by decision authority at work, was associated with an increased risk of depression during the follow-up period in male and female employees, even after adjusting for pre-baseline psychiatric diagnoses, background sociodemographic factors, and other factors related to the working environment | Pro: large sample size reduces bias due to selection and attrition  Cons: Interindividual variations in exposure levels within particular occupations cannot be measured with JEM; patients register capture only severe cases of depression; no validation studies for depression diagnosis in the Swedish patient registers available |
| Kim et al. (2020) [87] | CES-D (Korean version) | Categorised | Men: High job demand HR (95%-CI): 1.49 (1.38; 1.61)**; Insufficient job control HR (95%-CI): 1.16 (1.03; 1.30); Job insecurity 1.63 (1.48; 1.79)**; Organizational injustice: HR (95%-CI): 1.17 (1.06; 1.30)*; Discomfort in an organizational climate HR (95%-CI): 1.54 (1.42; 1.67)**  Women: High job demand HR (95%-CI): 1.32 (1.17; 1.49)**; Organizational injustice: HR (95%-CI): 1.39 (1.20; 1.61)**; Discomfort in an organizational climate HR (95%-CI): 1.35 (1.19; 1.53)** | age, center, marital status, education, income, AUDIT, smoking, BMI, total KOSS score, type of working hours, shift work, average work time | The results suggest gender and age differences in the relationship between occupational stress and incident depressive symptoms. | Selection bias |

^b^none (e.g, bivariate analysis, only), sociodemographic (e.g., age, gender, nationality, marital status, occupational status, education, income), work-related (e.g., working hours, night shift, full- or part-time position), health-related (e.g., self-rated health, depression in the past, health risks like smoking and protective factors like physical activity), private (e.g., private strains), others; *p<0.05; **p<0.01, ***p<0.001
