# Supplementary material for: The Relationship Between Work-Related Stress and Depression: A Scoping Review
Source: Public Health Rev. 2024 May 1;45:1606968. doi: 10.3389/phrs.2024.1606968 (PMC11094281; doi:10.3389/phrs.2024.1606968)
Supplement: Supplementary file 3 [file Table3.docx]

**Supplementary Table S3 Frequency of usage of different measurements of work-related psychosocial stress in included studies, Scoping review on the relationship between work-related stress and depression (six continents, 1999-2022) (N=125)**

| **Frequency of use [%]** | **Scale [Ref.]** | **Version** | | | | | **Reference number of publication** |
| --- | --- | --- | --- | --- | --- | --- | --- |
|  |  | **original** | **long** | **short** | **modified/ combined** | **Name/ Measurement** |  |
| 33/125  (26.4%) | Effort-Reward Imbalance (ERI) [33] | **X** |  |  |  | Original 23 item version with efforts (six items), rewards (11 items) and overcommitment (six items) | [99], [100], [101], [102], [103] |
|  |  | **X** |  |  | **c** | Original 23 item version with efforts (six items), rewards (11 items) and overcommitment (six items) | [98], [63], [74] |
|  |  |  | **X** |  | **c** | Efforts (five items), rewards (11 items) and overcommitment (six items) | [109], [155] |
|  |  |  | **X** |  |  | French validated version (23 items): Six items were related to effort, eleven to reward and six to overcommitment | [118], [119] |
|  |  |  | **X** |  |  | Chinese version (23 items) consisting of three subscales: effort (six items), reward (11 items) and overcommitment (six items) | [40], [44], [46], [53] |
|  |  |  | **X** |  | **c** | Japanese version (46 items) with efforts (six items), rewards (11 items) and overcommitment (29 items) | [76] |
|  |  |  | **X** |  |  | Japanese version (23 items) of the effort-reward imbalance questionnaire consisting of three subscales: effort (six items), reward (11 items) and overcommitment (six items) | [71] |
|  |  |  | **X** |  | **m** | ERI questionnaire consists of two reliable and valid subscales: ‘efforts’ (six items), ‘rewards’ (11 items) | [104] |
|  |  |  | **X** |  | **m/c** | 17-Items: ‘efforts’ (six items), ‘rewards’ (11 items) | [105] |
|  |  |  | **X** |  | **m** | The 17-item Korean version: ‘efforts’ (six items), ‘rewards’ (11 items) | [81] |
|  |  |  | **X** |  | **m/c** | ERI questionnaire consists of two subscales: ‘efforts’ (five items), ‘rewards’ (11 items) | [136] |
|  |  |  |  | **X** |  | Validated short version of ERI (Siegrist et al. 2009) efforts (three items), rewards (seven items) and overcommitment (six items) | [107], [108] |
|  |  |  |  | **X** | **m** | Validated short version of ERI (Siegrist et al. 2009) efforts (three items), rewards (seven items) without overcommitment | [77] |
|  |  |  |  | **X** | **m** | Chinese short version of ERI consisting of three items for the effort scale and seven items for the reward scale | [45] |
|  |  |  |  | **X** | **m** | Turkish version of the short ERI questionnaire (ERI-SV) (three items ‘effort’, seven items ‘reward’) | [90] |
|  |  |  |  | **X** | **m** | Abbreviated version of ERI used in the SHARE-study covering ‘efforts’ (two items) and ‘rewards’ (five items) | [132] |
|  |  |  |  | **X** | **m/c** | Abbreviated version used in the SHARE-study covering efforts (two items) and rewards (five items) | [130] |
|  |  |  |  | **X** | **m/c** | Effort-reward imbalance, two items measuring ‘effort’, and five items assessing ‘reward’ at work | [144], [161] |
|  |  |  |  | **X** | **m/c** | Work load was measured by a subscale of the short version of ERI | [106] |
|  |  |  |  | **X** | **m/c** | To measure effort-reward imbalance, two measuring ‘effort’, and five assessing ‘reward’ at work were included (four items in J-STAR) | [162] |
| 10/125  (8.0 %) | Job-Demand-Control (JDC) [32] | **X** |  |  | **m/c** | Japanese version of the JDC model: psychological demands (five items) and job control measured by two subscales- “skill discretion” (four items) and “decision authority” (two items) | [76] |
|  |  |  |  | **X** |  | Shortened Swedish version of the JDC-questionnaire: job demands (four items) and job control (six items) | [125] |
|  |  |  |  | **X** |  | Swedish version of the JDC-questionnaire/ Imbalance between work-related demands (work pace, deadlines, time pressure) and control (decision latitude) | [122] |
|  |  |  |  | **X** | **m** | Objective assessment of JDC (three questions on demands, two question on control) by external rater | [138] |
|  |  |  |  | **X** |  | Three items for psychological demand and the combination of two equally weighted scales of six and three items measuring skill discretion and decision authority respectively for job control | [154] |
|  |  |  |  | **X** | **c** | Three items measuring demands and three items measuring control | [144] |
|  |  |  |  | **X** | **m** | Job strain defined as a combination of job control (four items on decision authority) & job demands (three items on stress, time and level of concentration) measured by job exposure matrix  based on the Swedish Work Environment Surveys (1997–2013) | [128] |
|  |  |  |  | **X** | **m/c** | Only ‘control’ with two items | [130], [161], [162] |
| 7/1257/125  (5.6 %) | Job Demands-Control-Support (JDC-S)  [186] |  | **X** |  |  | Karasek’s extended JDC-S-model: 15 items for job control, four for job demand, six for social support at work | [73] |
|  |  |  |  | **X** |  | Four items on job demands (overload, hours at the office, hours at home, extra activities), two items on job control (control over flexibility in work hours and number of hours), two items on support (by coworkers and organization) | [133] |
|  |  |  | **X** |  | **m** | Workload (six items, similar to those of the job demands scale of JCQ); Job control (seven-item scale similar to the decision authority scale of the JCQ); social support (eight items) | [54] |
|  |  |  |  | **X** |  | Demands measured by three items (work pace, conflicting demands, enough time) from the Demand-Control-Support Questionnaire | [131] |
|  |  |  |  | **X** |  | Four items on demands and six items of support | [126], [127] |
|  |  |  | **X** |  | **m** | FIT-instrument as a screening instrument for the JDC-S(Richter, Hemmann, Merboth, Fritz, Hänsgen & Rudolf, 2000), captures six items of job demand and seven items of control | [105] |
|  |  |  |  | **X** | **m/c** | Job demands: (workload and assaults): subscale of the short version of the Effort-Reward Imbalance Questionnaire  Job resources: were assessed by the degree of social support, shared values and leadership climate  Social support (four Items) and shared values (four Items) were measured by an adapted German version of the Organizational Check-up Survey (Leiter & Maslach, 2000) by Beerlage, Hering, and Springer (2007) | [106] |
| 22/125  (17,6 %) | Job Content  Questionnaire (JCQ)  [184] | **X** |  |  | **c** | Full version of the JCQ to assess work stress in five dimensions: skill discretion (six items), decision authority (three items), psychological demands (five items), job insecurity (three items), supervisors/co-work social support (eight items) | [136] |
|  |  |  | **X** |  | **m** | Full Job Demand- Control-Support model based on JCQ-questions  job demands (five items), job control (nine items) and worksite social support (eight items) | [58] |
|  |  |  | **X** |  | **m** | Demand (five items) & control (skill discretion (six items) & decision authority (three items)) & support items of JCQ (supervisor support (four items) & co-worker support (four items) | [96] |
|  |  |  | **X** |  | **m /c** | Japanese version  Demand scale  Control scale  Support scale | [72] |
|  |  |  |  | **X** | **m** | Job demands and control, supervisor support and colleague support were obtained from a self-administered questionnaire | [64] |
|  |  |  |  | **X** | **m** | Job control (five questions), psychological demands (two questions), social support (three questions) | [152] |
|  |  |  |  | **X** | **m** | Decision latitude (15 items), work demands (four items), work social support (six items) in a self-administered questionnaire | [121] |
|  |  |  |  | **X** | **m** | 12 item short scale of the JCQ in six dimensions (skill discretion, decision authority, psychological demands, job insecurity, physical exertion, social support from supervisors and coworkers) | [93], [134], [135] |
|  |  |  |  | **X** | **m** | Job content plateau stress was also assessed using the Cohen et al., 1983 measure, this time inquiring specifically about stress associated with ‘‘career situation two (lack of challenge or responsibility)’’ | [146] |
|  |  |  | **X** |  | **m** /c | Job demands (eight items), job control (eight items) | [98] |
|  |  |  | **X** |  | **m** | Job demands (six items), job control (10 items) | [156] |
|  |  |  | **X** |  | **m** | Job demands (five items), job control (nine items) | [114] |
|  |  |  |  | **X** | **m** | Job demands (three items), job control (nine items) | [113] |
|  |  |  | **X** |  | **m** | Japanese version  Demand scale (five items)  Control scale (nine items) | [61] |
|  |  |  |  | **X** | **m** | Quantitative workload derived from the JCQ, work contents from the Experienced Job Resource Scale, eight items of physical work environment, Three items of job resources derived from the Dentist Experienced Job Resource Scale | [115] |
|  |  |  |  | **X** | **m** | Swedish Shortened version of the DCQ of the JCQ: Demand (five items), decision authority (four items) | [124] |
|  |  |  |  | **X** | **m** | Two demand subscales (psychological demands & emotional demands) and six control Items (skill discretion, decision authority and Macro-decision latitude) subscales of the new Job Content  Questionnaire (JCQ) 2.0 | [153] |
|  |  |  |  | **X** | **m** | Job Strain: the items measuring job demands (three items) and job control (nine items), derived from the Job Content Questionnaire + work unit + occupational title | [116] |
|  |  |  |  | **X** | **m** | Job demands were measured using a three-item scale from the Occupational Stress Questionnaire, developed by the Finnish Institute of Occupational Health (Elo, Leppänen, Lindström & Ropponen, 1992), job control was measured using nine items from the Job Content Questionnaire (Karasek, 1985) | [117] |
|  |  |  |  | **X** | **m/c** | Job insecurity (four items) | [57] |
| 7/125  (5.6 %) | Brief-Job  Stress  Questionnaire (BJSQ) [187] | **X** |  |  |  | 57 Items of work stressors (work overload and personal relations, psychosomatic responses to stress, social support and depressed mood) | [59] |
|  |  |  |  | **X** | **m** | Japanese version with three subscales: job quantitative overload (three items), job control (three items) and support from supervisors and coworkers (six items) | [60] |
|  |  |  |  | **X** | **m** | 12-item abbreviated version (job stressors subscale) | [62] |
|  |  |  |  | **X** | **m** | 17 of 57 items on psychological qualitative and quantitative workload, physical work load, stressors of personal relations in a workplace, stressors of working environment, job control, skill utilization, aptitude for job, and worth working | [75] |
|  |  |  |  | **X** | **m** | Job demands (three Items), job control (three Items), social support from supervisors, co-workers and family/friends (three items) | [67], [68], [70] |
| 2/125  (16.0 %) | 21 items from the  Child Care Worker Job Stress Inventory  [188] |  |  | **X** |  | Six items from the Job Demands subscale, five items  related to Job Specific Demands for teachers, five items from the Job Resources subscale, and  five items from the Job Control subscale | [143] |
|  |  |  |  |  | **X** | Two aspects of job stress (lack of resources and control) were assessed with the respective subscales of the modified version of CCW-JSI  Job Control subscale assesses perceived degree of control over various aspects of work  The Job Resources subscale assesses perceived availability of work resources (rewards) | [129] |
| 2/125  (1.6 %) | Copenhagen Psychosocial Questionnaire (‘‘COPSOQ’’) [189] |  |  | **X** | **m** | Dimensions: quantitative demands, possibilities  for development and influence at work | [97], [111] |
| 1/125  (0.8 %) | Job Stress Scale (JSS) | **X** |  |  |  | In the model proposed by Karasek [185], this scale was originally developed in Sweden, for evaluation of occupational stress: 17 questions, covering demand(five items), control (six items) and social support (six items) | [160] |
| 1/125  (0.8 %) | Brief Stress Scale (BSS) | **X** |  |  |  | BSS was developed for assessing stress in Taiwan (Mental Health Foundation 2008); includes six items covering various work stress situations and job characteristics (e.g., work control, workload, workplace relationships) | [95] |
| 1/125  (0.8 %) | Psychosocial Leave-Behind Questionnaire |  |  |  | **X** | 15-item index derived from Karasek’s job strain scale  15-items describe two factors: job stress (six items)  and job satisfaction (nine items) | [147] |
| 1/125  (0.8 %) | A shortened stress evaluation tool (ASSET)  [190] | **X** |  |  |  | Eight subscales of job stressors: work relationships, job nature, overload, job control, job security, resources/communication, work-life balance, pay and benefits | [39] |
| 1/125  (0.8 %) | Self-conducted Measurement of psychosocial work characteristics | **X** |  |  |  | Six psychosocial work characteristics: quantitative demands, influence at work, possibilities for development, social support from supervisors, social support from coworkers and job insecurity | [112] |
| 1/125  (0.8 %) | Self-conducted Measurement |  |  |  | **X** | Psychological demands (seven items)  Decision latitude (six items)  Supervisor support for work and family issues (ten items)  Workplace flexibility, or the policies and practices that aid employees in meeting their work, family, and personal responsibilities (nine items)  Co-worker support (three items)  Job insecurity five variables | [151] |
| 9/125  (7.2 %) | The National Institute for Occupational  Safety and Health Generic Job Stress Questionnaire (NIOSH  GJSQ) [164] |  |  | **X** | **m** | Including the following measures of Generic Job Stress Questionnaire (GJSQ) developed by NIOSH: role conflict, role ambiguity, job control, social support from coworkers, quantitative workload, variance in workload | [78] |
|  |  |  |  | **X** | **m** | Quantitative workload (four items), responsibility for others (four items), role conflict (four items), role ambiquity (five items) | [159] |
|  |  |  |  | **X** | **m/c** | Japanese version, which was developed by the NIOSH, measuring job overload (11 items), job control (16 items), role ambiguity (six items), role conflict (eight items), supervisor support (four items), coworker support (four items) | [57] |
|  |  |  |  | **X** | **m** | Japanese version measuring interpersonal- (each eight items of intra- and intergroup-) conflict | [56] |
|  |  |  |  | **X** | **m** | Japanese version without role conflict and job future ambiguity (seven job stressors and buffers) | [55] |
|  |  |  |  | **X** | **m** | Japanese version of the NIOSH Generic Job Stress  Questionnaire (GJSQ): four subscales to assess perceived individual level occupational stress (job control, quantitative workload, role conflict and role ambiguity) and three measures of social support (from supervisors, from co-workers and from family or friends) according to the DC/S model for the assessment of perceived individual level occupational stress | [65] |
|  |  |  |  | **X** | **m** | Quantitative workload, job control, role conflict, role ambiguity and social support (from supervisors and co-workers) | [66] |
|  |  |  |  | **X** | **m** | Quantitative workload, variance in workload, cognitive demand, job control, intragroup conflict, intergroup conflict, role ambiguity, role conflict, social support from supervisor, social support from coworker, social support from family/friend, non-work activity, and self-esteem | [69] |
|  |  |  |  | **X** | **m/c** | Social support dimension | [74] |
| 8/125  (6,4 %) | Korean occupational stress scale (KOSS) [165] | **X** |  |  |  | Eight subscales (43 items) from the most popular job stress measurement tools such as JCQ, ERI, NIOSH and OSI and a qualitative study: difficult physical environment (three items), high job demand (eight items), insufficient job control (five items), inadaequate social support (four items), job insecurity (six items), organizational injustice (seven items), lack of reward (six items), discomfort in occupational climate (four items). | [79] |
|  |  |  |  | **X** | m | Seven subscales (24 items), including job demand (four items), insufficient job control (four items), interpersonal conflict (three items), job insecurity (two items), organizational system (four items), lack of reward (three items) and workplace environment (four items) | [80], [84-87] |
|  |  |  |  | **X** | **m** | Three of the seven dimensions of KOSS (17 items): job demand (eight items), job control (five items), interpersonal conflict (four items) | [82], [83] |
| 1/125  (0.8 %) | Job Burden-Capital Matching Model [166] | X |  |  |  | Including workload based on effort dimension of the ERI-model (six items), psychological demand based on the demand dimension of the JCQ (four items) and capital based on JCQ and ERI questions (autonomy (two items), skills (three items), social support (two items), feedback (six items), work stability (two items), work prospect(two items) and personality (three items) based on over-commitment of ERI | [47] |
| 3/125  (2.4 %) | Perceived Stress Scale [167] |  |  |  | **X** | Perceived Stress Scale (PSS-10)  Psychological stress: Ten items designed to measure the degree to which situations in one’s life are appraised as stressful | [120] |
|  |  |  |  |  | **m/c** | Hierarchical plateau stress was measured with a modified version of Cohen, Kamarck and Mermelstein’s (1983) four-item Perceived Stress Scale | [146] |
|  |  |  |  |  | **X** | The abbreviated PSS scale used in this study has five of the 14 items | [149] |
| 1/125  (0.8 %) | Perceived Work Stress Scale (PWSS) |  |  |  | **X** | Seven Items to reflect perceptions of overall work-related stress or adapted from the Perceived Stress Scale [167] | [145] |
| 2/125  (1.6 %) | Perceived Stress Questionnaire (PSQ)  [168] | X |  |  |  | PSQ is a self-assessment-based instrument for recording subjective perceived stress | [97], [123] |
| 2/125  (0.8 %) | 5-item Clergy Occupational Distress Index (CODI) [169] | **X** |  |  |  | CODI measures congregational demands including the feeling of loneliness and isolation | [142], [148] |
| 1/125  (0.8 %) | Dentists’ Experienced Job Resources Scale [170] in combination with job demands | X |  |  |  | DEJRS specifically aimed at identifying and investigating resources in dentistry including craftsmansship, professional contacts, long-term and immediate results. Three items of Karasek’s job demands were included, additionally. | [115] |
| 1/125  (0.8 %) | Teacher Stress Inventory [171] |  | **X** |  | **X** | Modified version of the Teacher Stress Inventory, assessed 41 commonly occurring stressful situations occurring within the past year | [163] |
| 2/125  (1.6 %) | Taiwanese  Nurse Stress Checklist (TNSC) [172] |  |  |  |  | Job stress measured by the 43-item Taiwanese  Nurse Stress Checklist (TNSC) | [91], [92] |
| 1/125  (0.8 %) | Chinese  Nurse Stress Checklist [172] | **X** |  |  |  | 47 items in four subscales : Personal responses (17 items), work concerns (13 items), competency (11 items), incompleteness of personal arrangements (six items) | [94] |
| 1/125  (0.8 %) | Expanded Nursing Stress Scale (ENSS) [173] |  | **X** |  |  | Contains 57 items including nine subscales (death and dying, conflict with physicians, inadequate preparation, problems with peers, problems with supervisors, workload, uncertainty concerning treatment, patients and their families, and discrimination) | [89] |
| 1/125  (0.8 %) | Workplace bullying [174] | **X** |  |  | **c** | Negative Acts Questionnaire (NAQ) containing 22 items referring to direct and indirect behaviors of bullying | [72] |
| 1/125  (0.8 %) | WFC [191] | **X** |  |  | **c** | WFC questionnaire with each four question on work to family and family to work conflict | [136] |
| 1/125  (0.8 %) | The short-form Occupational Stress Indicator [192] |  |  | **X** |  | 20 items: consists six dimensions of potential sources of job stress:  including Factors Intrinsic to Job (e.g., having to work very long hours), Role in the Organization (e.g., managing or supervising the work of other people), Relationships with Others (e.g., lack of social support by people at work), Career and Achievement (e.g., unclear promotion prospects), Organizational Structure and Climate (e.g., characteristics of the organization’s structure and design), and Home/Work Interface (e.g., home life with a  partner who is also pursuing a career) | [43] |
| 1/125  (0.8 %) | Occupational Stress Inventory-revised (OSI-R) [193] | **X** |  |  |  | Chinese Version of OSI-R measures with 10 items each: role overload, role insufficiency, role ambiguity, role boundary, responsibility | [48] |
| 1/125  (0.8 %) | Wheaton’s chronic stress scale [194]. | **X** |  |  |  | Five items on perceived stress including items like ‘my supervisor is always watching what I do work’ or ‘my work is boring and repetitive’ | [139] |
| 1/125  (0.8 %) | Work pressure subscale from the Work Environment Scale [195] |  |  | **X** |  | Nine-item version of the Work pressure subscale;  The questionnaire assesses the degree to which the workplace is characterized by high job demands, long work hours, and frequent deadline | [140] |
| 1/125  (0.8 %) | House and Rizzo's Work Stress Scale  [196] | **X** |  |  |  | Chinese version with 11 Items. House and Rizzo's Work Stress Scale measures organizational and leadership practices | [41] |
| 2/125  (1.6 %) | Mental Stressor Investigation  Questionnaire [197] |  |  | **X** |  | A short form of the scale with 36 items that addressed two factors:  ship environment, and relations between work and interpersonal (Heavy workloads, intense time pressures, latitude in decision-making, occupational risks, lack of support from co-workers) | [42] |
| 1/125  (0.8 %) | Uehata Stress Questionnaire [198] | **X** |  |  | **c** | 14 questions concerning work-related  stress | [63] |
| 1/125  (0.8 %) | The Hospital Consultants Job Stress and Satisfaction Questionnaire [199] |  |  | **X** |  | Questionnaire adopted from HCJSSQ : 21 questions on sources of stress, 13 factors on factors contributing on sources of stress, 16 questions on methods adopted for coping stress | [158] |
| 1/125  (0.8 %) | Trier Inventory of Chronic Stress [200] |  |  | **X** |  | From a total of nine subscales only work overload, pressure to succeed, work dissatisfaction, excessive demands and lack of acceptation was used | [110] |
| 1/125  (0.8 %) | Cai and Jiang 3-item scale for the Chinese labor force [201] | **X** |  |  |  | Three –item scale asking whether work makes physically and mentally exhausting, gives a lot of pressure and whether employees losing interest in this job day after day | [50] |
| 1/125  (0.8 %) | DASS-21 stress scale [202] | **X** |  |  |  | Self-perceived stress measured with the DASS (depression anxiety stress)-21 stress scale | [88] |
| 1/125  (0.8 %) | Devilliers, Carson and Leary (DCL) stress scale [203] | **X** |  |  |  | The items included (1) Having to deal with disturbed individuals, (2) Conflicts not being settled, and (3) Lack of positive feedback from supervisors within the organization and from unpredictable patients. | [157] |
| 1/125  (0.8 %) | Work-life balance stress | **X** |  |  |  | Self-constructed question: ‘To what extent you perceive stress to keep your work-life balance?’ | [52] |
| 1/125  (0.8 %) | Working hours | **X** |  |  |  | Average weekly working hours and weekend | [109] |
| 2/125  (1. %) | Work stress, self contructed | **X** |  |  |  | Self-constructed instrument including five dimensions: (1) work overload; (2) role ambiguity; (3) role conflict; (4) limited work-related decision-making authority; (5) client diability | [141] |
|  |  |  |  | **X** |  | A short form of the scale with 27 items addressed two factors:  ship environment, and working relationships | [51] |
| 1/125  (0.8 %) | Emotional demand and occupational resources | **X** |  |  |  | 10 item emotional demand scale with four aspects: need to show positive or negative emotions (three items), emotional sympathy (three items), demand for sensitivity (three items), emotional suppression (two items); occupational resources measured with job autonomy scale (four items), job satisfaction (four items), job security (two items) | [137] |
| 1/125  (0.8 %) | Scale developed by China’s Labor Force Dynamic Survey (CLDS) | **X** |  |  |  | CLDS three –item scale asking participants (1) work is exhausting, (2) work is always stressful, (3) losing interest in job more and more | [49] |
| 1/125  (0.8 %) | The Job Role Quality Scale | **X** |  |  |  | Job stress assessed using the Job Role Quality Scale a 21-item measure that captured women’s positive and negative experiences at work, including opportunity for advancement, recognition  and supervisor support | [150] |
| 1/125  (0.8 %) | Social support scale of  Caplan, Cobb, French, VanHarrison, and Pinneau’s [204] |  |  | **X** | **m/c** |  | [155] |

Abr.: c= combined with other instrument(s); m = modified
