# Supplementary material for: The Relationship Between Work-Related Stress and Depression: A Scoping Review
Source: Public Health Rev. 2024 May 1;45:1606968. doi: 10.3389/phrs.2024.1606968 (PMC11094281; doi:10.3389/phrs.2024.1606968)
Supplement: Supplementary file 4 [file Table4.docx]

**Supplementary Table S4 Frequency of usage of different measurements of depression/ depressive symptoms/ depressiveness in included studies,** **Scoping review on the relationship between work-related stress and depression (six continents, 1999-2022)** (N=125)

| **Frequency of use**  **[%]** | **Scale/ Measurement** | **version** | | | | | **Reference number of publication** |
| --- | --- | --- | --- | --- | --- | --- | --- |
|  |  | **original** | **long** | **short** | **modified/ combined** | **name** |  |
| **57/125**  **(45.6 %)** | **Center for Epidemiological Survey-Depression Scale (CES-D)**  **[176]** | **X** |  |  |  | Center for Epidemiological Survey-Depression Scale | [55], [63], [73-75], [82], [84], [95], [118], [119], [129], [139], [140], [145], [146], [149], [150], [159], [162], [163] |
|  |  | **X** |  |  |  | Center for Epidemiological Survey-Depression Scale, Chinese | [39], [40], [42], [44], [46], [48], [51] |
|  |  | **X** |  |  |  | Center for Epidemiological Survey-Depression Scale, Japanese | [56], [57], [62], [69], [70-72], [76-78] |
|  |  | **X** |  |  |  | Center for Epidemiological Survey-Depression Scale, Korean | [79], [85-87] |
|  |  | **X** |  |  |  | Center for Epidemiologic Studies Depression Scale, Turkish | [90] |
|  |  |  |  | **X** |  | Center for Epidemiological Studies–Depression Scale (shorter Iowa form, 11 items) | [96] |
|  |  |  |  | **X** |  | Center for Epidemiological Survey-Depression Scale, 15 items | [98] |
|  |  |  |  | **X** |  | Center for Epidemiological Studies–Depression Scale, 10 items | [43], [52], [141] |
|  |  |  |  | **X** |  | Center for Epidemiological Survey-Depression Scale, 8 items | [144], [147], [161] |
|  |  |  | **X** |  |  | Center for Epidemiologic Studies Depression Scale (21 borderline version by Cho and Kim to distinguish between non-depression and depression) | [81] |
|  |  |  |  |  | **X** | Center for Epidemiological Survey-Depression Scale, 16 items (4 positive items removed) | [137] |
|  |  |  |  | **X** |  | ADS-K (German version of CES-D, short form with 15 items) | [102], [103], [105] |
|  |  |  |  | **X** | **X** | CES-D (11-item abridged adaptation) | [155] |
|  |  |  |  | **X** | **X** | CES-D (modified form, 7 items) | [133] |
| **16/125**  **(12.8 %)** | **Beck Depression Inventory (BDI)**  **[177]** | **X** |  |  |  | Beck Depression Inventory | [109], [113], [158] |
|  |  |  |  | **X** |  | Beck Depression Inventory (short form with 13-Items) | [114], [115] |
|  |  | **X** |  |  |  | Beck Depression Inventory-II | [89], [91], [108-110], [117], [157] |
|  |  | **X** |  |  |  | Beck Depression Inventory-V | [83], [99], [100], [104] |
| **12/125**  **(9.6 %)** | **Patient Health Questionnaire (PHQ)**  **[214]** | **X** |  |  |  | Patient Health Questionnaire PHQ-8 | [54] |
|  |  | **X** |  |  |  | Patient Health Questionnaire PHQ-9 | [45], [47], [68], [80], [106], [136] [142], [148], [153], [156] |
|  |  | **X** |  |  |  | Patient Health Questionnaire PHQ-9, Japanese version | [67] |
| **5/125**  **(4.0 %)** | **Zung’s Self-Rating Depression Scale (SDS)**  **[179]** | **X** |  |  |  | Zung’s Self-Rating Depression Scale | [58] |
|  |  | **X** |  |  |  | Zung’s Self-Rating Depression Scale, Chinese version | [41] |
|  |  | **X** |  |  |  | Zung’s Self-Rating Depression Scale, Japanese version | [61], [65], [66] |
| **5/125**  **(4.0 %)** | **ICD Diagnosis** | **X** |  |  |  | ICD-10 | [97], [111], [116], [128] |
|  |  | **X** |  |  |  | ICD-9 | [138] |
| **4/125**  **(3.2 %)** | **EURO-D depression scale [180]** | **X** |  |  |  | EURO-D depression scale | [130], [132], [161], [162] |
| **3/125**  **(2.4 %)** | **Hopkins Symptom Checklist (SCL-90) [212]** |  | **X** |  |  | SCL Core Depression scale (SCL-CD6) short version with 6 items of the brief subscale from the Hopkins Symptom Checklist (SCL-90) | [125], [126], [131] |
| **3/125**  **(2.4 %)** | **Taiwanese Depression Questionnaire (TDQ) [205]** | **X** |  |  |  | Taiwanese Depression Questionnaire (TDQ) | [92-94] |
| **3/125**  **(2.4 %)** | **DSM Diagnosis** | **X** |  |  |  | DSM-IV  SCID-I Structured Clinical Interview for the DSM-IV Axis I | [55], [154], [158] |
| **2/125**  **(1.6 %)** | **A 5-item scale constructed *ad hoc*. In past 4 weeks how often have you: felt sad, lost self-confidence, encountered insurmountable difficulties, been able to get going or daily life, been affected due to emotional problems? 1-5 (never to always)** |  |  |  |  | No specific name | [49], [50] |
| **1/125**  **(0.8 %)** | **Depression Anxiety Stress Scales (DASS-21) [202]** | **X** |  |  |  | Depression Anxiety Stress Scales (DASS-21) | [88] |
| **1/125**  **(0.8 %)** | **Self-reported doctor’s diagnosis.** |  |  |  |  | Self-reported doctor’s diagnosis.  Answer to question: Has any doctor or health professional ever said that you have depression?  Has any doctor or health professional ever said that you have depression? | [160] |
| **1/125**  **(0.8 %)** | **Brief Job Stress Questionnaire (BJSQ) [187]** | **X** |  |  |  | Depressed mood measured by Brief Job Stress Questionnaire (BJSQ) | [59] |
| **1/125**  **(0.8 %)** | **Brief Symptom Inventory-18 (BSI-18) [206]** |  |  | **X** |  | 6-item depressive symptoms subscale from Brief Symptom Inventory-18 (BSI-18) | [140] |
| **1/125**  **(0.8 %)** | **Common Mental Disorder Questionnaire (CMDQ) [207]** | **X** |  |  |  | Common Mental Disorder Questionnaire (CMDQ) | [111] |
| **1/125**  **(0.8 %)** | **General Health Questionnaire (30-item version) (GHQ-30) [208]** | **X** |  |  |  | General Health Questionnaire (30-item version) (GHQ-30) | [63] |
| **1/125**  **(0.8 %)** | **Harvard National Depression Screening Day Scale [209]** | **X** |  |  |  | Harvard National Depression Screening Day Scale | [120] |
| **1/125**  **(0.8 %)** | **Kessler 6 (K6) scale [210]** | **X** |  |  |  | Kessler 6 (K6) scale | [64] |
| **1/125**  **(0.8 %)** | **Mental Health Inventory five-item of the 36-item Short-Form Health Survey [211]** |  |  | **X** |  | Mental Health Inventory five-item of the 36-item Short-Form Health Survey | [112] |
| **1/125**  **(0.8 %)** | **Symptom Checklist-core depression (SCL-CD6) [212]** | **X** |  |  |  | Cutoff for major depression was obtained by using the Major Depression Inventory (MDI) as index of validity | [131] |
| **1/125**  **(0.8 %)** | **Montgomery – Asb scaleerg depression rating scale+ Hamilton scale [181]** | **X** |  |  |  | Montgomery – Asb scaleerg depression rating scale+ Hamilton scale | [123] |
| **1/125**  **(0.8 %)** | **Pearlin 9-item questionnaire [213]** | **X** |  |  |  | Pearlin 9-item questionnaire | [122] |
| **1/125**  **(0.8 %)** | **Patient Health Questionnaire (PHQ-9) [215]** | **X** |  |  |  | Patient Health Questionnaire (PHQ-9) based on the DSM-IV | [54] |
| **1/125**  **(0.8 %)** | **RAND health depression screening tool [178]** | **X** |  |  |  | RAND health depression screening tool | [143] |
| **1/125**  **(0.8 %)** | **Screening scale with items developed by Fukisawa et al. [215]** |  |  |  |  | A 5-item screen was derived from the Self-rating Depression Scale and the Hospital Anxiety and Depression Scale, using the Composite International Diagnostic Interview as the external criterion. | [60] |
| **1/125**  **(0.8 %)** | **Self-Rating Depression Scale by Sirodff [216]** |  |  |  |  |  | [53] |
| **1/125**  **(0.8 %)** | **State-Trait Depression Scales [217]** | **X** |  |  |  | State-Trait Depression Scales (German version, 10 items) | [101] |
| **1/125**  **(0.8 %)** | **The Hamilton Rating Scale for Depression (HRSD-24) [218]** | **X** |  |  |  | The Hamilton Rating Scale for Depression for the doctor’s diagnosis of the severity of depressive symptoms | [108] |
| **1/125**  **(0.8 %)** | **indicator including two domains of depression in the last**  **month:**  **feeling ‘‘down, depressed, or hopeless’’ and ‘‘little interest”**  **or pleasure in doing things’’.** |  |  |  |  |  | [151] |
